# Evaluating the feasibility of gene replacement strategies to treat *MTRFR* deficiency

**DOI:** 10.1242/dmm.052120

**Published:** 2025-06-02

**Authors:** Samia L. Pratt, Mariana Zarate-Mendez, Lidiia Koludarova, Sonja Jansson, Mikko Airavaara, Irena Hlushchuk, David Coleman, Caleb Heffner, Rita Horvath, Brendan J. Battersby, Robert W. Burgess

**Affiliations:** ^1^ The Jackson Laboratory, Bar Harbor, ME 04609, USA; ^2^ Neuroscience Ph.D. Program, Graduate School of Biomedical Science, Tufts University, Boston, MA 02111, USA; ^3^ Department of Clinical Neurosciences, John Van Geest Centre for Brain Repair, University of Cambridge, CB2 0PY Cambridge, UK; ^4^ Institute of Biotechnology, HiLIFE, University of Helsinki, 00014 Helsinki, Finland; ^5^ Drug Research Program, Faculty of Pharmacy, University of Helsinki, 00014 Helsinki, Finland

**Keywords:** *C12ORF65*, Leigh syndrome, CMT6, Mitochondrial translation, MRPL58, Behr's syndrome

## Abstract

Mitochondrial translation release factor in rescue (MTRFR) catalyzes a termination step in protein synthesis, facilitating release of the nascent chain from mitoribosomes. Pathogenic variants in *MTRFR* cause *MTRFR* deficiency and are loss-of-function variants. Here, we tested gene replacement as a possible therapeutic strategy. A truncating mutation (K155*) was generated in mice; however, homozygotes die embryonically whereas mice heterozygous for this K155* allele are normal. We also generated transgenic strains expressing either wild-type human *MTRFR* or a partially functional *MTRFR*. Despite dose-dependent phenotypes from overexpression *in vitro*, neither transgene caused adverse effects *in vivo*. In K155* homozygous mice, the wild-type *MTRFR* transgene completely rescued the phenotype with only one copy present, whereas the mutant transgene rescued less efficiently. Detailed evaluation of mice rescued with the wild-type *MTRFR* transgene revealed no abnormalities. In human induced pluripotent stem cell (hiPSC)-derived knockdown neurons, mitochondrial phenotypes were corrected by AAV9-mediated delivery of *MTRFR*. Thus, we find no toxicity from truncated gene products or overexpression of *MTRFR in vivo*, and expression of *MTRFR* corrects phenotypes in both mouse and hiPSC models.

## Teaser

**Summary:** There are currently no treatments for *MTRFR* deficiencies; however, after using genetic approaches in mice and a human induced pluripotent stem cell model, we conclude that *MTRFR* deficiencies are a good candidate for gene therapy treatments.

## INTRODUCTION

Mitochondrial translation release factor in rescue (*MTRFR*), previously referred to as chromosome 12 open reading frame 65 (*C12ORF65*), is a ubiquitously expressed nuclear gene encoding a mitochondrial class 1 release factor. Recessive pathogenic variations in *MTRFR* cause rare mitochondrial disorders described as Behr's syndrome, Leigh syndrome and Charcot-Marie-Tooth disease type 6 (CMT6), often including optic and peripheral neuropathy, as described below.

Mitochondria contain their own genome, mitochondrial DNA (mtDNA). In mammals, mtDNA is a strictly maternally inherited circular genome encoding 13 proteins, all of which help form four of the complexes required for oxidative phosphorylation (Kasamatsu et al., 1971). mtDNA also encodes 22 transfer RNAs (tRNAs) and two ribosomal RNAs (rRNAs) that are crucial for the translation of proteins. All are transcribed as a polycistronic mtRNAs (Kummer and Ban, 2021; Ott et al., 2016) that are subsequently processed to liberate individual messenger RNAs (mRNAs), tRNAs and rRNAs. Mitochondrial mRNAs are then translated into proteins on mitochondrial-specific ribosomes (mitoribosomes) within the organelle. Mitoribosomes contain two rRNA molecules and one tRNA, which is a structural component of the mitoribosome. In addition, mitoribosomes contain 82 nuclear encoded proteins, 36 of which have no cytosolic or bacterial homologs (Amunts et al., 2015; Brown et al., 2014; Englmeier et al., 2017; Greber et al., 2014). Mitochondrial translation varies from canonical cytoplasmic translation, as protein synthesis occurs in a manner more similar to translation of bacterial polycistronic RNAs.

**Fig. 1.**
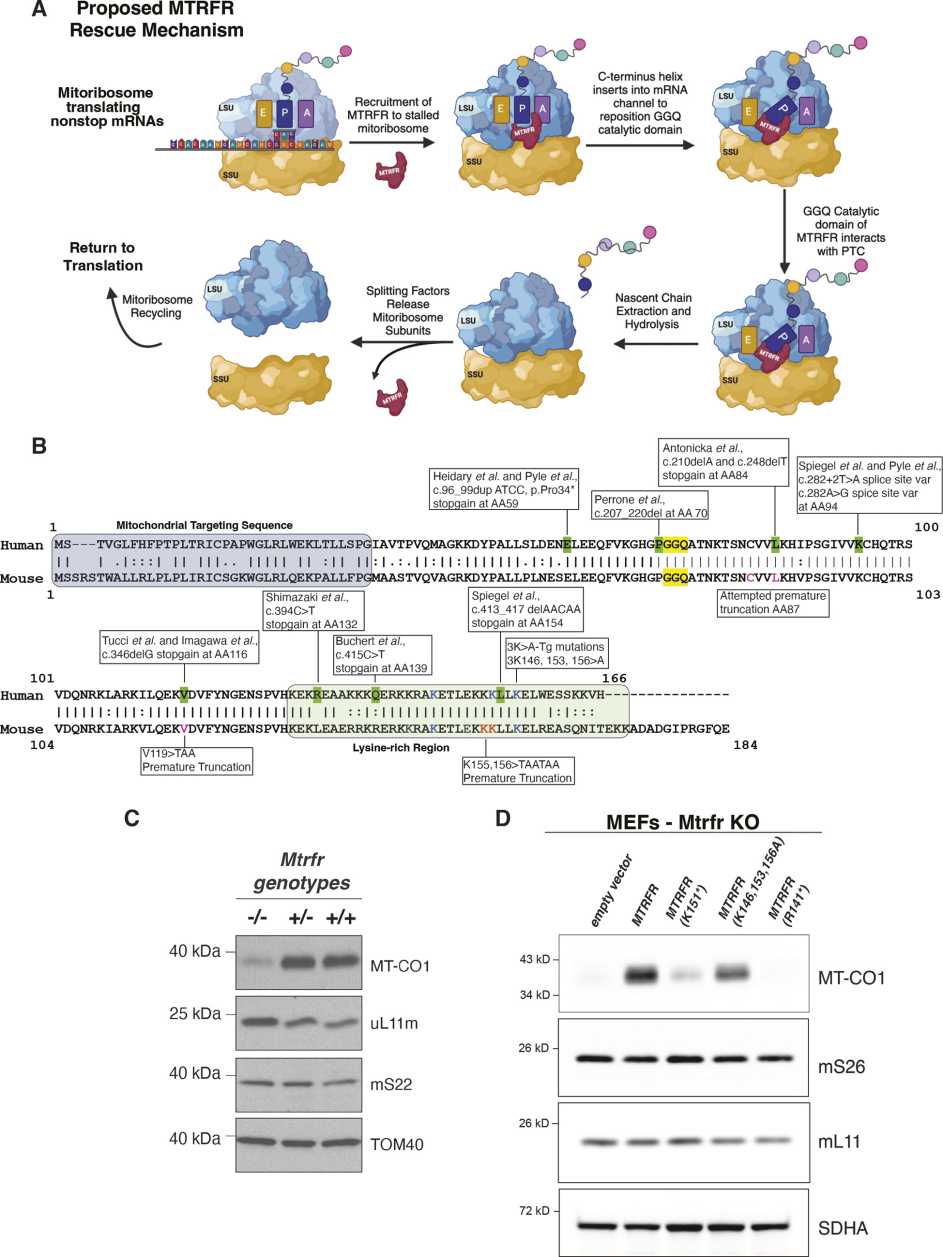
**Structure and function of MTRFR.** (A) The mechanism of MTRFR rescuing stalled mitochondrial translation is schematized. ‘GGQ catalytic domain’ is referring to the glycine-glycine-glutamine catalytic domain of MTRFR, and ‘PTC’ is referring to the peptidyl-transferase center of the mitoribosome. Created in BioRender by Pratt, S. L. (2025). https://BioRender.com/nzmjvti. This figure was sublicensed under CC-BY 4.0 terms. (B) Protein sequence alignment of human and mouse MTRFR. ‘|’ denotes an exact match between the sequences, ‘:’ denotes most highly conserved substitution, and ‘.’ denotes semiconserved substitution. The mitochondrial targeting sequence (MTS) is based on a prediction of the human protein using UniProt. Human *MTRFR* codes for a protein 166 amino acids long, and green highlight indicates homozygous or compound heterozygous pathogenic variants observed in patients. Yellow highlight indicates the highly conserved glycine-glycine-glutamine (GGQ) domain. Mouse *Mtrfr* encodes a protein 184 amino acids long; different colors depict variants used in our models. Red indicates premature truncation at amino acids 155 and 156 (K155,156*); blue indicates three lysines mutated to alanines at amino acids 146, 153 and 156; pink denotes mutations previously attempted to induce a premature truncation mouse model (amino acids 87 and 119). (C) Immunoblot of whole-cell lysates from mouse embryonic fibroblasts (MEFs) with the indicated *Mtrfr* genotypes. The steady-state abundance of MT-CO1 is a robust indicator for the defect in mitochondrial protein synthesis and is reduced in homozygous cells. The other three proteins in this blot [uL11m (also known as MRPL11), mS22 (also known as MRPS22) and TOM40 (also known as TOMM40)] are all nuclear-encoded genes that function within the mitochondria, showing that differences in *Mtrfr* do not affect cytosolic protein synthesis. (D) Immunoblot of whole-cell lysates from *Mtrfr*-knockout (KO) MEFs following retroviral transduction with an empty vector, and wild-type and mutated human *MTRFR* cDNAs with the indicated genotypes. The truncation at R141 results in a protein that is inactive, truncation at K151 results in a protein that retains a low level of activity, and substitution of lysines K146, K153 and K156 for alanines results in a protein that retains moderate activity.

MTRFR is one of four known class 1 mitoribosomal release factors – mitochondrial translation release factor 1 (MTRF1), mitochondrial translation release factor 1A [MTRF1A; also known as mitochondrial translation release factor 1 like (MTRF1L)], mitochondrial ribosomal protein L58 (MRPL58) and MTRFR – that could play a role in mitochondrial protein synthesis termination (Kummer et al., 2021) (Fig. 1A). Intriguingly, MTRFR is both structurally and functionally homologous to bacterial alternative release factor B (ArfB) (Carbone et al., 2020; Huter et al., 2017; Kogure et al., 2012; Ng et al., 2022; Richter et al., 2015), which terminates protein synthesis on bacterial non-stop ribosome complexes. These four release factors all contain three major domains: (1) an amino-terminal mitochondrial targeting sequence (MTS), (2) a type 1 release factor domain with a conserved catalytic glycine-glycine-glutamine (GGQ) motif and (3) a C-terminal alpha helix (Amunts et al., 2015; Richter et al., 2010) (Fig. 1B). The α helical C-terminus is required for interaction with the mitoribosome, while the GGQ motif is responsible for catalyzing the release of the nascent chain from the tRNA in the P-site (Gagnon et al., 2012; Ng et al., 2022).

MTRFR is proposed to resolve non-stop mitoribosomes complexes as it does not contain the structural motif required to recognize stop codons (Ng et al., 2022). Non-stop mitoribosome complexes form during translation of mRNAs that lack stop codons (Saurer et al., 2023) (Fig. 1A). In animals, eight of the 13 mitochondrial mRNA open reading frames do not include a stop codon and have a tRNA flanking at the 3′ end instead of a traditional stop codon (Anderson et al., 1981; Ng et al., 2022). Precise tRNA processing by a mitochondrial RNase P complex followed by post-transcriptional polyadenylation would generate a stop codon in these mitochondrial mRNAs. Deep-sequencing approaches of mRNAs from translating mitoribosomes, however, have revealed low-level errors in mitochondrial tRNA processing and polyadenylation, which generate mRNAs that lack a stop codon (Ietswaart et al., 2024; Ng et al., 2022). Further, there do not appear to be any quality control mechanisms to prevent translation initiation on mitochondrial mRNAs with aberrant 3′ ends, such as lacking a stop codon (Ietswaart et al., 2024; Ng et al., 2022). In the absence of a stop codon, a translating mitoribosome could stall, leaving the mRNA channel empty past the A-site and the nascent chain attached to the tRNA in the P-site. Prolonged mitoribosome stalling leads to mitochondrial and cellular stress (Richter et al., 2013; Richter et al., 2019).

To terminate protein synthesis on these non-stop mRNAs, MTRFR is recruited through unknown mechanisms. The positively charged, lysine-rich C-terminal α helix inserts into the mRNA channel of the mitoribosome, and, if there is stalling, the GGQ motif interacts with the peptidyl-transferase center, catalyzing the extraction of the nascent peptide chain (Fig. 1A) (Ng et al., 2022). Splitting factors then release the two mitoribosome subunits so that they can be recycled (Desai et al., 2020). It is hypothesized that MTRFR acts only as a rescue release factor for non-stop mitochondrial mRNA complexes, as it lacks the characteristic domain that recognizes mitochondrial mRNA stop codons that is conserved among other release factors (Ayyub et al., 2020; Desai et al., 2020; Nadler et al., 2022; Ng et al., 2022). Disease-associated variants within MTRFR are usually premature truncations of the protein, resulting in peptides lacking or with shortened C-termini, which are essential for interacting with the mitoribosomes and sensing the non-stop stalls (Fig. 1B).

Because mitochondria depend on MTRFR for maintenance of proper translation levels (Antonicka et al., 2010; Buchert et al., 2013; Desai et al., 2020; Suomalainen and Battersby, 2018), loss of MTRFR is highly detrimental to the cell (Antonicka et al., 2010; Nishihara et al., 2017; Schubert Baldo and Vilarinho, 2020; Wesolowska et al., 2015). When *MTRFR* is deficient or mutated, mitochondrial translation remains stalled, leading to a severe and disabling neurological disease with spastic paraparesis, optic and peripheral neuropathy diagnosed as Behr's syndrome (Buchert et al., 2013; Pyle et al., 2014; Shimazaki et al., 2012; Spiegel et al., 2014), Leigh syndrome (Heidary et al., 2014) and CMT6 (Tucci et al., 2014). Patients with homozygous and compound heterozygous autosomal recessive pathogenic variants in *MTRFR* usually present in early childhood with progressive neuronal degeneration. Symptoms include progressive optic atrophy, peripheral motor neuropathy, distal muscle weakness and spastic paraparesis (Fang et al., 2017; Olimpio et al., 2021; Pyle et al., 2014). In more severe cases, patients have also presented with a variable degree of ataxia and intellectual disabilities. All of these symptoms have a significant negative impact on the life of the patients and families. There are 11 reported pathogenic variants in the *MTRFR* gene that have been described (Perrone et al., 2020), and, despite the heterogeneity in symptom severity, there is a proposed correlation between genotype and phenotype (Spiegel et al., 2014). Patients with later truncations closer to the C-terminus of the protein have less severe symptoms than those of patients who have earlier truncations closer to the GGQ motif. Unfortunately, there are currently no gene-specific treatments for patients with *MTRFR* variants, only palliative care.

*MTRFR* deficiency is one of a large group of disorders linked to defects in mitochondrial translation (Suomalainen and Battersby, 2018). Rare recessive diseases, like Behr's syndrome and *MTRFR* deficiency stemming from *MTRFR* variants, tend to be good candidates for gene replacement therapies such as adeno-associated virus (AAV) delivery of rescuing wild-type complementary DNAs (cDNAs) (Di Meo et al., 2017; Hanaford et al., 2022). A different therapeutic modality that would deliver wild-type *MTRFR* or bolster expression of partially functional alleles could also be considered for treatment; but, given the recent successes with AAV treatments in rare neurodegenerative diseases and the early-stage nature of other options, virally mediated gene replacement is a viable near-term strategy. However, before any therapy bolstering *MTRFR* expression can be implemented, a number of factors must be addressed. These include possible detrimental effects of gene overexpression, possible dominant negative activities of prematurely truncated proteins, and whether the human *MTRFR* sequence can indeed fully rescue function in cross-species preclinical studies. It has been shown that overexpression of mitochondrial proteins can generate toxic effects on overall mitochondrial function by overwhelming the mitochondrial translation machinery, and it is important for us to test this in our mammalian system. Here, we show that loss-of-function mouse mutations are fully rescued by a human *MTRFR* transgene, that there are no adverse effects due to presence of the truncated protein, and that overexpression of a human transgene does not cause any discernable phenotype in mice. Therefore, *MTRFR* deficiency and recessive loss-of-function pathogenic variants in *MTRFR* are good candidates for a gene therapy-mediated gene replacement strategy.

## RESULTS

### Mutations in *Mtrfr*

The human *MTRFR* gene is located on chromosome 12 and consists of three exons that encode a protein 166 amino acids in length. Patients presenting with *MTRFR* deficiency and related disorders are homozygous or compound heterozygous for pathogenic variants in *MTRFR* that are often premature truncations (Fig. 1B) (Antonicka et al., 2010; Buchert et al., 2013; Heidary et al., 2014; Perrone et al., 2020; Pyle et al., 2014; Shimazaki et al., 2012; Spiegel et al., 2014; Tucci et al., 2014). Alignment of the human and mouse protein sequence shows the over 78% similarity and conservation between the species. The mouse *Mtrfr* gene is located on chromosome 5 and consists of three exons that encode a protein of 184 amino acids. Initial attempts to introduce patient variants in mouse *Mtrfr* included truncations at amino acids 87 or 119 (V116 in humans). However, these alleles were embryonically lethal as homozygotes and are likely complete loss-of-function alleles as they are missing the entire C-terminal alpha helical region. Based upon the proposed phenotype to genotype correlation (Ng et al., 2022; Spiegel et al., 2014), we explored patient-associated variants closer to the C-terminus of the protein to create possible partial loss-of-function alleles. In *in vitro* assays, the function of MTRFR was assessed using the steady-state abundance of MT-CO1 protein levels as a proxy for mitochondrial translation. Mouse embryonic fibroblasts (MEFs) with no functional *Mtrfr* had deficits in mitochondrial translation as seen by a decrease in the production of MT-CO1, whereas cytoplasmic translation levels remained unchanged (Fig. 1C). Proteins like TOM40 that are translated by cytoplasmic ribosomes but reside and function in the mitochondria also remained unchanged, indicating that there is no influence on translation of genes encoded in the nucleus or import into the mitochondria (Fig. 1C). Our work in MEFs also showed that the severity of the truncation influences the extent of the translation deficits, recapitulating the genotype to phenotype correlation seen in MTRFR-deficient patients (Fig. 1D; Fig. S1) (Buchert et al., 2013; Ng et al., 2022; Spiegel et al., 2014). Truncations at amino acid 141 resulted in little to no detectable MT-CO1, whereas a truncation at 151 resulted in reduced, but detectable, levels of MT-CO1, indicating retention of some activity. In addition, altering three lysines (at positions 146, 153 and 156) to alanines in the C-terminal domain also produced a protein with only partially impaired function in this assay (Fig. 1D).

We therefore engineered two premature stop codons at positions 155 and 156 (changing AAA codons to TAA codons), referred to as the *Mtrfr*^K155*^ mouse model (Fig. 1B) (Spiegel et al., 2014). Mice heterozygous for the K155* mutation show no phenotype. However, this allele results in embryonic lethality in homozygotes. Timed matings and embryo harvests were performed, which revealed a failure to recover homozygotes after embryonic day (E)12 (Table 1). At E10.5 and E11.5, homozygous embryos were recovered at the expected frequency; however, at E12.5, E13.5 and E14.5, no homozygous embryos were recovered. Thus, even using an allele that should retain partial activity based on extrapolation from the human truncation series and cell culture assays (Ng et al., 2022), there was no postnatal survival, indicating that mice are more sensitive to loss of *Mtrfr* than humans.

**Table 1.** MTRFR deficiency causes embryonic lethality in mice homozygous for the K155* premature truncation

	Number (percentage) at timepoint				
	E10.5	E11.5	E12.5	E13.5	E14.5
Wild type (+/+)	2 (22.2%)	13 (26.5%)	6 (24%)	3 (14.3%)	3 (33.3%)
Heterozygote (−/+)	5 (55.6%)	29 (59.2%)	19 (76%)	18 (85.7%)	9 (66.6%)
Homozygote (−/−)	2 (22.2%)	7 (14.3%)	0	0	0
Total harvested	9	49	25	21	9
Chi-squared value (*P*-value)	0.00 (*P*=1.000)	2.807 (*P*=0.2458)	11.083 (**P=0.0039)	10.255 (**P=0.0059)	4.500 (*P*=0.0154)

Mice homozygous for the K155* premature truncation were not recovered after embryonic day (E)11.5. Chi-squared analysis was done with two degrees of freedom. **P<0.01.

### *MTRFR* overexpression

**Fig. 2.**
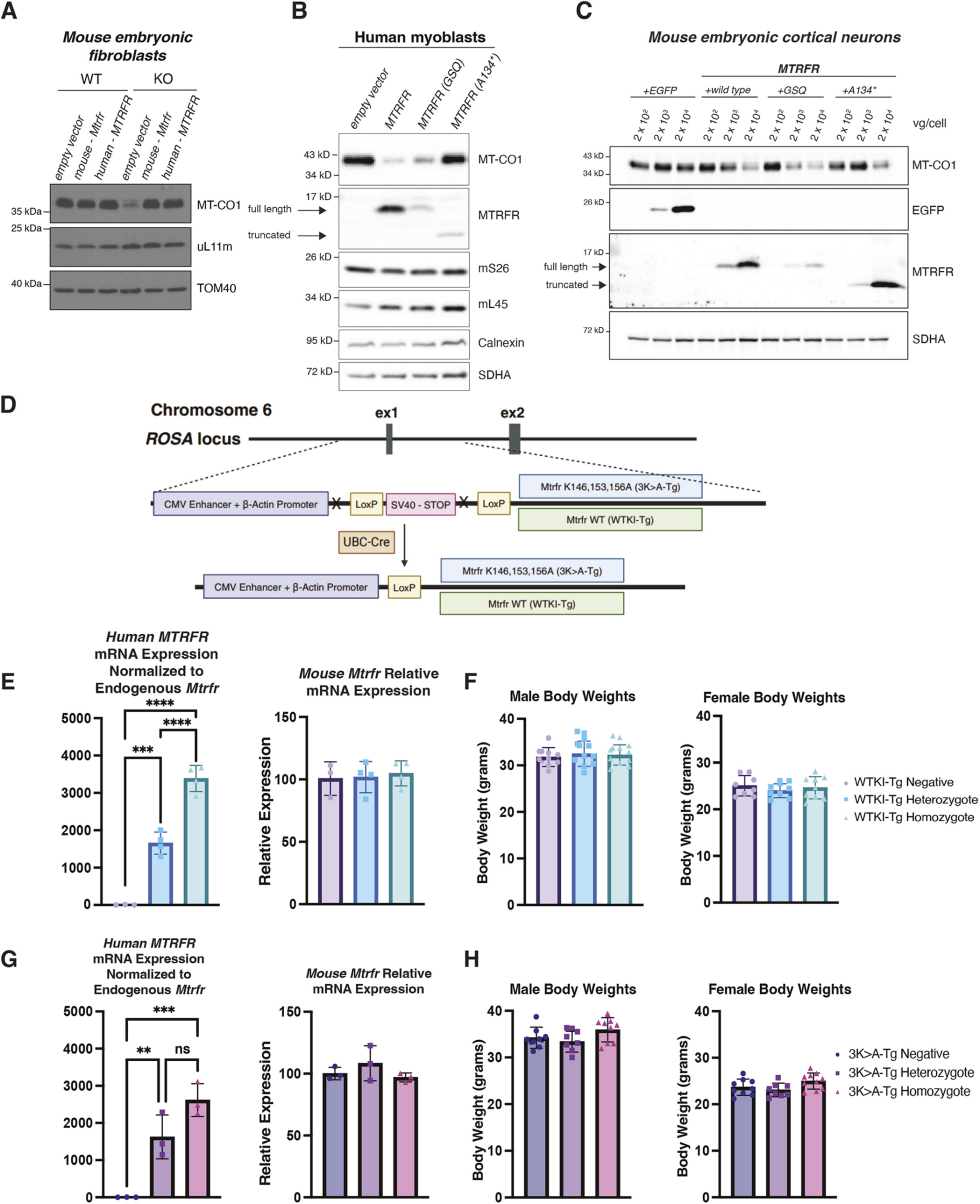
**Overexpression of MTRFR and phenotyping.** (A) Immunoblot of whole-cell lysates of *Mtrfr*-KO and wild-type MEFs following retroviral transduction with wild-type cDNA of mouse *Mtrfr* or human *MTRFR* shows that both rescue the deficit in mitochondrial translation (reduced MT-CO1 levels). (B) Immunoblots of wild-type human myoblasts treated with wild-type or mutated human *MTRFR* cDNAs. Expression of wild-type and MTRFR with a mutated catalytical site (GSQ) led to reduced steady-state abundance of MT-CO1. (C) Immunoblot of mouse embryonic cortical neuron cultures treated with an EGFP control vector or vectors expressing wild-type and mutated human *MTRFR* cDNAs at 2×10^2^, 2×10^3^ and 2×10^4^ viral genomes/cell (vg/cell). High levels of MTRFR led to reduced steady-state abundance of MT-CO1. (D) A schematic of the transgene constructs inserted into the *Rosa26* locus on mouse chromosome 6. Wild-type human gene (WTKI-Tg) or mutated human gene (3K>A-Tg) cDNAs were inserted into the genome driven by the CMV enhancer and beta-actin promoter. A LoxP-flanked stop cassette was deleted using a ubiquitous *Cre* transgenic strain to create constitutively expressing substrains used for these studies. ‘ex1’ and ‘ex2’ denote the first and second exon of the *Rosa26* locus, respectively. WT, wild type. Created in BioRender by Pratt, S. L. (2025). https://BioRender.com/25hjouh. This figure was sublicensed under CC-BY 4.0 terms. (E) RT-qPCR was done using RNA extracted from spinal cord samples of wild-type (*n*=3), heterozygous (*n*=4) and homozygous (*n*=4) transgenic mice using primers specific to the mouse or human genes. The expression of human *MTRFR* was normalized to the endogenous expression of *Mtrfr*; endogenous *Mtrfr* expression was also measured using a different primer pair. (F) Body weights for males and females were measured at 9 months of age and did not differ with genotype. Mouse numbers were as follows: WTKI-Tg-negative, males *n*=11, females *n*=8; WTKI-Tg-heterozygote (het), males *n*=12, females *n*=9; WTKI-Tg-homozygote (hom), males *n*=12, females *n*=9. (G) RT-qPCR was done using RNA extracted from spinal cord samples of wild-type, heterozygous and homozygous transgenic 3K>A mice (*n*=3/genotype). The expression of transgenic *MTRFR* was normalized to the endogenous expression of mouse *Mtrfr*, and endogenous *Mtrfr* expression was also measured. (H) Body weights for both males and females were measured at 9 months of age and did not differ with genotype. Mouse cohorts were as follows: 3K>A-Tg-negative, males *n*=10, females *n*=9; 3K>A-Tg-het, males *n*=8, females *n*=8; 3K>A-Tg-hom, males *n*=10, females *n*=10. Error bars show s.d. Statistical analysis was performed using one-way ANOVA. ns, not significant; **P<0.01, ***P <0.001, ****P<0.0001.

With the end goal of assessing whether *MTRFR* deficiency is a good candidate for gene therapy approaches to replace the mutated gene, we assessed possible rescue and the effects of overexpression in MEFs, myoblasts, cortical neurons and mice. Human and mouse *Mtrfr* were packaged for retroviral transduction of MEFs. We found that both the human and mouse genes were able to rescue mitochondrial translation deficits in *Mtrfr*-knockout (KO) cells (Fig. 2A). However, there was a threshold of tolerability of overexpression. In human myoblasts, we saw that overexpression of human wild-type *MTRFR* led to a reduction in MT-CO1 protein levels (Fig. 2B), showing a detrimental effect on mitochondrial translation. A decrease in MT-CO1 levels was also seen when the myoblasts were treated with partially functional *MTRFR* with a mutation in the catalytic domain where the second glycine in the GGQ motif was mutated to serine (GSQ), but there was no decrease in MT-CO1 levels when the myoblasts were treated with nonfunctional *MTRFR* prematurely truncated at amino acid A134 (Fig. 2B). These data indicate that the effects on mitochondrial translation are stronger when there is overexpression of functional or partially functional MTRFR in the myoblasts. These effects were also only seen in mitochondrial protein synthesis, as levels of nuclear encoded proteins (mS26, mL45, calnexin and SDHA) remained unchanged (Fig. 2B).

The *MTRFR* gene was then packaged into an AAV9 capsid for delivery to mouse embryonic cortical neurons. Three *MTRFR* AAV9s were developed, one wild-type, one with a mutation in the catalytic domain (GSQ) and the third with a truncation at amino acid 134, as described above. The cortical neurons were treated with each AAV9 vector at a multiplicity of infection (MOI) of 2×10^2^, 2×10^3^ or 2×10^4^ viral genomes/cell. Treatment with an MOI of 2×10^2^ is benign, but higher doses of wild-type *MTRFR* caused a decrease in MT-CO1 levels. Similar results were obtained with both mutated *MTRFR* treatments, with even the amino acid 134 truncation showing effects at the highest MOI (Fig. 2C). These data indicate that there is a level of overexpression of MTRFR that is detrimental to the cell. Therefore, because expression of *MTRFR* in *Mtrfr*-KO MEFs rescued mitochondrial translation deficits, but there were harmful effects of overexpressing too much *MTRFR*, we next investigated the effects of overexpression and *MTRFR* rescue *in vivo* in our K155* mice.

To do this, we created two transgenic mouse lines expressing either the wild-type human *MTRFR* gene or a variant with three C-terminal lysines changed to alanines at amino acids 146, 153 and 156, disrupting the charge and coiling of the α helix at the C-terminus of the protein (Fig. 2D; Fig. S2). These transgenes were targeted to the *Rosa26* safe-harbor locus on mouse chromosome 6 (Fig. S2). We anticipated that the mutated 3K>A-Tg protein would retain some activity (Fig. 1C), based on the ability to partially rescue MT-CO1 translation (Fig. 1D), whereas the wild-type transgene (WTKI-Tg) protein should be completely functional, barring differences in the mouse to human sequence. These conditional knock-in transgenic mice were bred with a ubiquitous *Cre* transgene to delete the floxed stop cassette in the germline and produce constitutively expressing substrains that were used in all experiments described below.

**Fig. 3.**
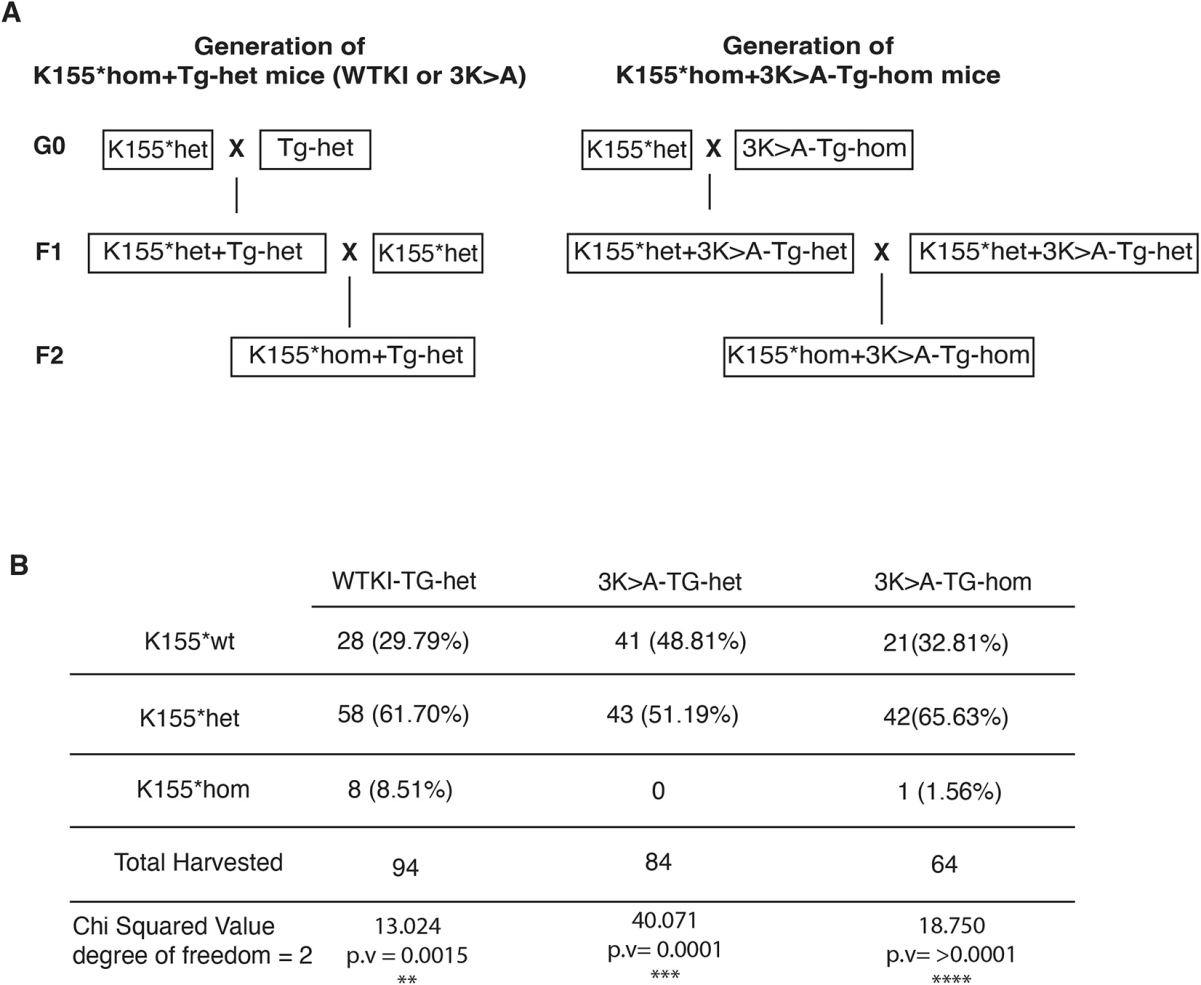
Rescue crosses of *Mtrfr*^K155*^ with wild-type or 3K>A *MTRFR* transgenes. (A) The breeding schematic for two generation crosses to test whether either transgene could rescue *Mtrfr*^K155*^ homozygous lethality (schematics of generation of K155*hom+WTKI-Tg-het and K155*hom+3K>A-Tg-het mice). Breeding schematic for the generation of K155*hom+3K>A-Tg-hom mice. (B) The number of WTKI-Tg-het mice (*n*=94) or 3K>A-Tg-het mice (*n*=84) that were K155 wild-type (wt)/het/hom were recorded, and a chi-squared analysis was performed to determine whether genotypes were being recovered at the expected rate. Although the WTKI-Tg rescued the homozygous K155* at a lower than predicted frequency, eight mice were recovered, whereas the 3K>A-Tg did not rescue. When the 3K>A-Tg was bred to produce homozygous transgenic mice (*n*=33), one K155* homozygote was rescued; however, that genotype was still under-represented. p.v, *P*-value. **P<0.01, ***P <0.001, ****P<0.0001.

To ensure that there was proper expression of the transgenes, reverse transcription quantitative PCR (RT-qPCR) from spinal cord mRNA was done from mice that were either heterozygous (Tg-het), homozygous (Tg-hom) or Tg-negative for each transgene at 9 months of age. *MTRFR* transgene mRNA levels were normalized to endogenous *Mtrfr* mRNA expression, and, as expected, there was a stepwise increase whereby expression in the homozygotes was roughly double that of the heterozygotes for both the WTKI (Fig. 2E) and 3K>A (Fig. 2G) transgenes. There were no changes in the endogenous mouse *Mtrfr* mRNA levels, confirming that presence of the transgenes does not influence the expression of the endogenous gene (Fig. 2E,G). These results confirm robust expression of the human transgenes in mouse and in the spinal cord, which contains disease-relevant cell types.

Having confirmed expression of the transgenes, we next assessed whether this level of overexpression of *MTRFR* would be deleterious *in vivo*. A broad phenotyping panel was undertaken looking at body weight and histopathological examination of major organs and the primary tissues affected by *MTRFR* deficiency. There were no differences in male or female body weights with either transgene (Fig. 2F,H). Histology was also performed on heart, liver, kidney, spleen, medial gastrocnemius, whole eye, brain and spinal cord. Tissues were evaluated by a veterinary pathologist, and no remarkable abnormalities that correlated with genotype were observed (Table 2). Given that peripheral and optic nerves are targets for future gene therapy approaches, we also examined these tissues for possible effects of overexpression. Both optic nerves were collected and stained, as well as sciatic nerves and the motor and sensory branches of the femoral nerves, and no abnormalities were found with *MTRFR* overexpression (Table 2). Overexpression of MTRFR could overwhelm the capacity to import the protein to the mitochondria, leading to misfolding or aggregation in the cytoplasm. However, as confirmed by our veterinary pathologist, there were no signs of protein aggregation via Hematoxylin and Eosin (H&E) or ubiquitin staining in the spinal cord (Table 2). Thus, in all tissues examined, there were no adverse effects from overexpression of *MTRFR* achieved by these transgenes (wild-type and 3K>A) under these conditions with these levels of expression. These data support that gene replacement with the wild-type gene could be a viable therapeutic strategy and that there is no dominant negative effect from a partially functional gene product (3K>A).

**Table 2.** Histopathological analysis of MTRFR mice

Tissues harvested													
						Nervous tissue							
								Nerves					
					Muscle	Optic tissue							
	Heart	Liver	Kidney	Spleen	Medial gastrocnemius	Whole eye	Retina	Optic nerve	Sciatic nerve	Femoral motor nerve	Femoral sensory nerve	Brain	Spinal cord
WTKI-Tg-negative *n*=3 (1F, 2M)	−	rare EMH (*n*=1/3)	mild peripelvic mononuclear infiltrate (*n*=3/3)	−	rare shrunken myofibers (*n*=2/3)	remnant hyaloid artery (*n*=1/3)	−	−	−	−	−	−	−
WTKI-Tg-heterozygote *n*=4 (2F, 2M)	−	−	mild peripelvic mononuclear infiltrate (*n*=4/4)	−	−	remnant hyaloid artery (*n*=4/4)	−	−	−	−	−	−	−
WTKI-Tg-homozygote *n*=4 (2F, 2M)	−	rare EMH (*n*=2/4)	mild peripelvic mononuclear infiltrate (*n*=4/4)	−	rare shrunken myofibers (*n*=3/4)	remnant hyaloid artery (*n*=2/4)	−	−	−	−	−	−	−
3K>A-Tg-negative *n*=3 (1F, 2M)	−	−	rare mild peripelvic mononuclear infiltrate (*n*=2/3)	−	rare myofibers, centralized nuclei (*n*=3/3)	remnant hyaloid artery (*n*=3/3)	−	−	−	−	−	−	−
3K>A-Tg-heterozygote *n*=3 (2F, 1M)	−	rare EMH (*n*=1/3)	rare cytoplasmic vacuoles and infiltrate (*n*=3/3)	−	rare shrunken myofibers (*n*=2/3)	remnant hyaloid artery (*n*=2/3)	−	−	−	−	−	−	−
3K>A-Tg-homozygote *n*=3 (2F, 1M)	−	rare EMH (*n*=1/3)	rare cytoplasmic vacuoles and infiltrate (*n*=3/3)	−	rare myofibers, centralized nuclei (*n*=1/3)	remnant hyaloid artery (*n*=2/3)	−	−	−	−	−	−	−
K155wt *n*=3 (1F, 2M)	rare vacuoles (*n*=1/3)	rare EMH (*n*=1/3)	mild peripelvic mononuclear infiltrate (*n*=3/3)	lymphoid follicles (*n*=3/3)	rare shrunken myofibers, rare cytoplasmic vacuoles (*n*=3/3)	−	−	−	−	−	−	−	−
K155*het *n*=4 (2F, 2M)	rare vacuoles (*n*=1/4)	−	mild peripelvic mononuclear infiltrate (*n*=4/4)	lymphoid follicles (*n*=4/4)	rare shrunken myofibers (*n*=4/4)	−	−	−	−	−	−	−	−

Table showing histological analysis of WTKI-Tg mice (WTKI-Tg-negative *n*=3, WTKI-Tg-heterozygous *n*=4, WTKI-Tg-homozygous *n*=4), 3K>A-Tg mice (3K>A-Tg-negative *n*=3, 3K>A-Tg-heterozygous *n*=3, 3K>A-Tg-homozygous *n*=3), wild-type mice for the K155 truncation (K155wt; *n*=3) and heterozygous littermates (K155*het; *n*=4). All tissues were taken at 9 months of age. Abnormalities noted by a veterinary pathologist are listed, along with their frequency of occurrence. No abnormalities segregated with a particular genotype. EMH, extramedullary hematopoiesis; F, female; M, male.

### Transgenic rescue of the *Mtrfr*^K155*^ allele

Having confirmed that overexpression of either a wild-type or the 3K>A form of human *MTRFR* had no adverse effects in the mice, we next asked whether human *MTRFR* could rescue the embryonic lethality seen in the K155* homozygotes. To test this, we created an intercross in which the mice would carry one copy of one of the transgenes and potentially be homozygous for the K155* premature truncation (Fig. 3A). This breeding strategy produced wild-type, heterozygous and homozygous K155* mice in the endogenous mouse *Mtrfr* gene on chromosome 5, with or without the human *MTRFR* transgene on chromosome 6. Viable offspring from these crosses were genotyped postnatally to assess rescue. The WTKI-Tg was able to rescue the embryonic lethality of the K155*homozygotes. Out of 94 WTKI-Tg-het mice, 28 were *Mtrfr* wild-type, 58 were K155*het and eight were K155*hom. Although the presence of the WTKI-Tg was sufficient to rescue the embryonic lethality, the K155*hom+WTKI-Tg-het mice are still under-represented (chi-squared *P*=0.0015). Out of 84 3K>A-Tg-het mice, zero K155*hom mice were born, representing a significant absence of that genotype (chi-squared *P*=0.0001) (Fig. 3B). Given that the WTKI-Tg rescues with a single copy in the *Rosa26* locus and that the partially functional 3K>A-Tg transgene does not, we reconfigured the breeding scheme to attempt to rescue the K155* homozygotes with two copies of the 3K>A-Tg (Fig. 3A). Of 64 mice that genotyped as homozygous for the 3K>A-Tg, one *Mtrfr*^K155*^ homozygote was recovered (Fig. 3B). This genotype was still under-represented (chi-squared *P*≥0.0001); however, the surviving mouse was indistinguishable from control littermates. It was not studied in detail as it is an *n*=1 for this genotype, but its presence is consistent with a partially functional *MTRFR* allele being able to rescue at higher gene dosage.

### Rescue of *Mtrfr*^K155*^ with WTKI-Tg is complete in the peripheral nervous system

**Fig. 4.**
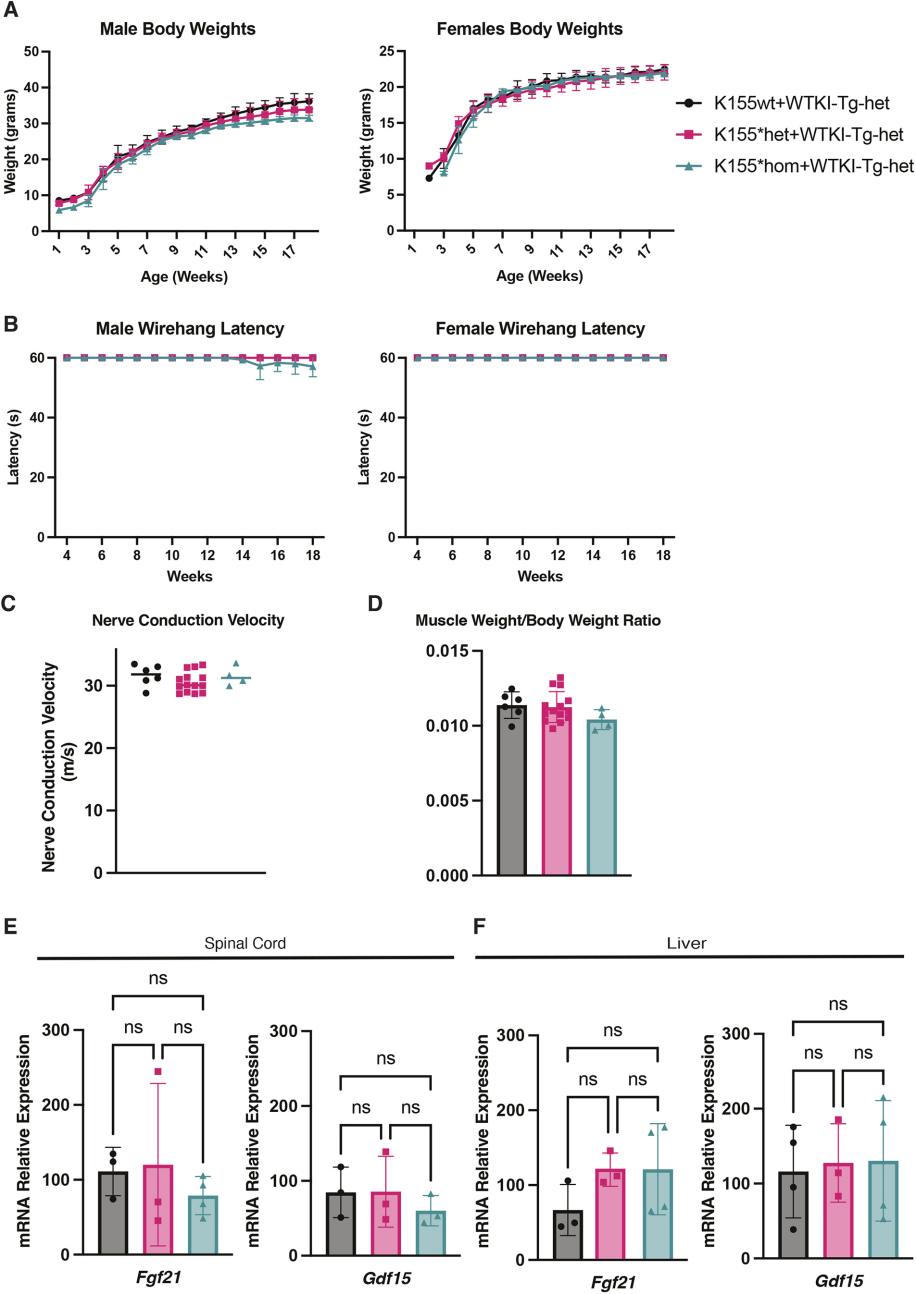
Phenotyping of WTKI-Tg-het rescue in K155wt/*het/hom mice. (A) Male and female body weights of K155wt/het/hom mice, all heterozygous for WTKI-Tg, were taken weekly from birth to 18 weeks of age. No differences between genotypes were found. (B) Wire hang latency to fall was measured for male and female mice from 4 weeks to 18 weeks to test muscle and grip strength. No differences between genotypes were found. (A,B) Males: K155wt *n*=5, K155*het *n*=14, K155*hom *n*=4. Females: K155wt *n*=9, K155*het *n*=12, K155*hom *n*=4. Statistical analysis was performed using a one-way ANOVA. (C) Sciatic motor nerve conduction velocity of K155wt/*het/hom mice rescued with heterozygous WTKI-Tg was measured at 18 weeks of age. No differences with genotype were found. (D) Muscle weight (medial gastrocnemius) to body weight ratio of K155wt/*het/hom mice rescued with heterozygous WTKI-Tg was measured. No differences with genotype were found. (C,D) K155wt *n*=6, K155*het *n*=14, K155*hom *n*=4. Statistical analysis was performed using a one-way ANOVA. There were no sex differences, and data were pooled. (E) RT-qPCR analysis of mRNA levels of the integrated stress response (ISR) target genes *Fgf21* and *Gdf15* in spinal cord from K155wt/*het/hom mice rescued with heterozygous WTKI-Tg. (F) ISR target gene expression in liver from K155wt/*het/hom mice rescued with heterozygous WTKI-Tg. No elevated expression was found in either tissue. (E,F) K155wt *n*=3, K155*het *n*=3, K155*hom *n*=4. Statistical analysis was performed using a one-way ANOVA. ns, not significant. There were no sex differences, and data were pooled. Error bars±s.d. in all panels.

To assess the completeness of the rescue of the K155*hom mice with heterozygous WTKI-Tg in tissues relevant to *MTRFR* deficiency, we completed a thorough analysis of peripheral nervous system phenotypes. Body weights from male and female mice were monitored weekly until terminal analysis at 18 weeks of age, and there was no significant difference in male or female K155*hom+WTKI-Tg-het body weights compared to those of their littermates that were either wild type or heterozygous for the K155* allele (Fig. 4A). Wire hang latency was also measured to assess possible deficits in grip strength or endurance, and again no significant differences were seen in either the male or female rescued homozygotes compared to their littermates (Fig. 4B). Sciatic motor nerve conduction velocity (NCV) was measured at 18 weeks, and again there was no difference between the genotypes, with only normal variation between cohorts (Fig. 4C). The triceps surae was dissected out and weighed to determine muscle weight to body weight ratio as an indicator of selective muscle atrophy; however, no differences were observed (Fig. 4D). Overall, the rescued *Mtrfr*^K155*^ mice showed no signs of peripheral nervous system dysfunction using tests that show differences in other mouse models of inherited peripheral neuropathy (Hines et al., 2023; Martin et al., 2023; Morelli et al., 2019, 2017; Murray et al., 2023; Spaulding et al., 2021, 2016; Tadenev et al., 2023).

The integrated stress response (ISR) is a stress pathway known to be activated in mitochondrial dysfunction and in other mouse models of inherited peripheral neuropathy (Anderson and Haynes, 2020; Forsstrom et al., 2019; Lehtonen et al., 2016; Mick et al., 2020; Spaulding et al., 2021). To test whether incomplete rescue of *Mtrfr* leads to activation of the ISR, RT-qPCR from liver and spinal cord samples was performed looking at two genes associated with the ISR in other mouse models of peripheral neuropathy: fibroblast growth factor 21 (*Fgf21*) and growth differentiation factor 15 (*Gdf15*). These are target genes of the transcription factor ATF4, the induction of which is an outcome of activation of the ISR. *Fgf21* and *Gdf15* were selected from the ISR pathway owing to their prominent induction in disorders related to mitochondrial dysfunction (Jennings et al., 2022; Montero et al., 2016; Poulsen et al., 2020; Spaulding et al., 2021). In both the liver and spinal cord samples, there was no increased expression of either *Fgf21* or *Gdf15*, indicating that these reporters of ISR activation were normal in these mutant mice (Fig. 4E,F) and supporting that the transgenic rescue of *Mtrfr*^K155*^ is complete for these tissues at these timepoints, aside from the under-representation of viable rescued pups, consistent with a persistent level of embryonic lethality. However, we also looked specifically for possible peripheral nerve histopathology and optic neuropathy phenotypes, as these are key features of patients with *MTRFR* deficiency.

### Peripheral nerve and retina histopathology in WTKI-Tg rescued mice

**Fig. 5.**
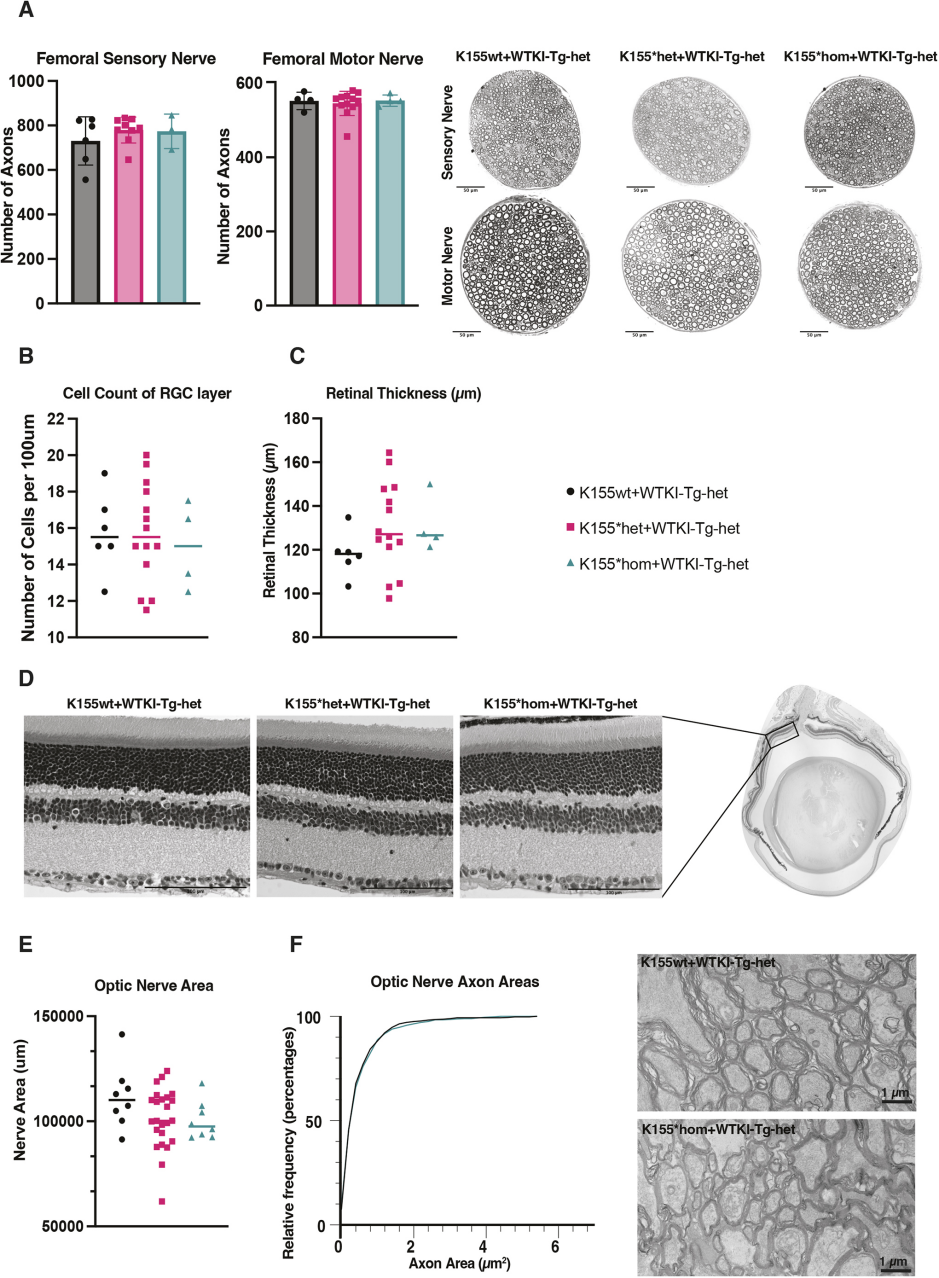
Rescue of K155* with heterozygous WTKI-Tg. (A) Axons from motor and sensory branches of femoral nerve were counted and did not differ by genotype. Representative images of each nerve in each genotype are shown. Femoral motor nerves: K155wt *n*=6, K155*het *n*=9, K155*hom *n*=3. Femoral sensory nerves: K155wt *n*=4, K155*het *n*=12, K155*hom *n*=4. There were no sex differences, and data were pooled. (B) Cells in the retinal ganglion cell (RGC) layer of the retina were counted in images from whole-eye histology. The data were normalized to number of cells per 100 µm of the RGC layer, and all counts were done adjacent to the optic nerve head. No differences with genotype were found. (C) The thickness of the retina from the base of the photoreceptor inner segments to the inner limiting membrane of the RGC layer was measured in the same retinal images, and no differences were found with genotype. (B,C) K155wt *n*=6, K155*het *n*=14, K155*hom *n*=4. (D) Representative images of retina histology for each genotype are shown. (E) The area of optic nerve cross-sections in each genotype was measured to assess possible optic nerve atrophy, but no differences were found. Mouse numbers were as follows: K155wt *n*=8, K155*het *n*=24, K155*hom *n*=8. (F) Using transmission electron microscopy, optic nerve axon diameters and myelin thicknesses for over 100 axons (103-136) were imaged per nerve and evaluated. Data are plotted as a cumulative histogram, showing that the distributions of axon sizes were not different with genotype. Optic nerves from K155wt (*n*=4) and K155*hom mice (*n*=4) were imaged. Statistical analysis was performed using one-way ANOVA (A-C,E) and Kolmogorov–Smirnov and Mann–Whitney tests (F). Graphs show mean±s.d. Scale bars: 50 µm (A), 100 µm (D) and 1 µm (F).

To substantiate the behavioral and physiological rescue we observed (Fig. 4), we also analyzed peripheral nerves by histopathology, as well as the retina and optic nerve, which are also severely affected in patients with *MTRFR* deficiency. The motor and sensory branches of the femoral nerve were dissected free, fixed and stained with Toluidine Blue, and the axon number, axon size and myelination of the nerves were assessed. Axons were counted from both the motor and sensory branches of the femoral nerve, and there were no signs of axon loss in either (Fig. 5A). The distribution of axon calibers was normal, and there were no signs of demyelination when the G-ratio (axon diameter/total fiber diameter of axon plus myelin) was analyzed (Fig. S3). Thus, consistent with the normal motor behavior and neurophysiology in rescued mice (Fig. 4), there were also no changes evident by histology.

Analyses of the retina and optic nerve were performed to evaluate possible optic neuropathy in the K155*hom+WTKI-Tg-het mice. Whole eyes were dissected free and fixed using paraformaldehyde (PFA) and then stained with H&E to evaluate retinal anatomy. The cell density in the retinal ganglion cell (RGC) layer adjacent to the optic nerve head was analyzed, and overall retinal thickness was determined. In the RGC, there was no change in the number of cells in any genotype, indicating no RGC loss (Fig. 5B). Because RGC axons make up the optic nerve, loss of cells in the RGC layer would contribute to optic nerve atrophy. We next quantified retinal thickness, measuring from the base of photoreceptor inner segments to the inner limiting membrane of the RGC layer. We did not see any differences in retinal thickness across the three genotypes, suggesting normal retinal cell number and anatomy (Fig. 5C). From the images of retinas from each genotype (Fig. 5D), there was no sign of disorganization or changes within the retinal layers. Overall, the retinal histology was indistinguishable between the genotypes and there were no signs of optic neuropathy.

We also evaluated the optic nerve itself. Nerves were isolated, and the right optic nerve was stained with Toluidine Blue to examine myelination, whereas the left optic nerve was stained with paraphenylenediamine (PPD) to detect any degenerating axons. The optic nerves stained with PPD showed no signs of dead or dying axons. The areas of the whole optic nerves were measured, and there was no significant difference between the genotypes (Fig. 5E). To look in detail at the individual axons in the optic nerves from *Mtrfr*^K155*^ homozygous mice rescued with one copy of the WTKI-Tg and *Mtrfr* wild-type mice with one copy of the transgene, the optic nerves were examined with transmission electron microscopy. From these images, the areas of over 100 axons from each optic nerve were analyzed, and the distribution of sizes was compared along with myelin thickness. There were no differences in the distribution of axon areas or myelination of the axons (Fig. 5F). Based on these data from optic nerve and retina, there are no signs of optic neuropathy in the *Mtrfr*^K155*^ mice when rescued with one copy of the WTKI-Tg.

Together, these data confirm that although a premature truncation of endogenous mouse *Mtrfr* gene causes embryonic lethality at ∼E12, this phenotype can be rescued with presence of a wild-type human *MTRFR* transgene. Although these homozygotes are still under-represented owing to persistent embryonic lethality, the mice that do survive postnatally show no detectable phenotype. The expression of wild-type human *MTRFR* in mouse does not lead to any adverse effects with either one or two copies of the *MTRFR* transgene. Similarly, there are no adverse effects from a truncated gene product (*Mtrfr*^K155*^ heterozygotes) or from expression of a semi-functional gene product (3K>A-Tg mice).

### AAV9 delivery of *MTRFR* rescues mitochondrial defect in an hiPSC-derived neuronal model of *MTRFR* deficiency

**Fig. 6.**
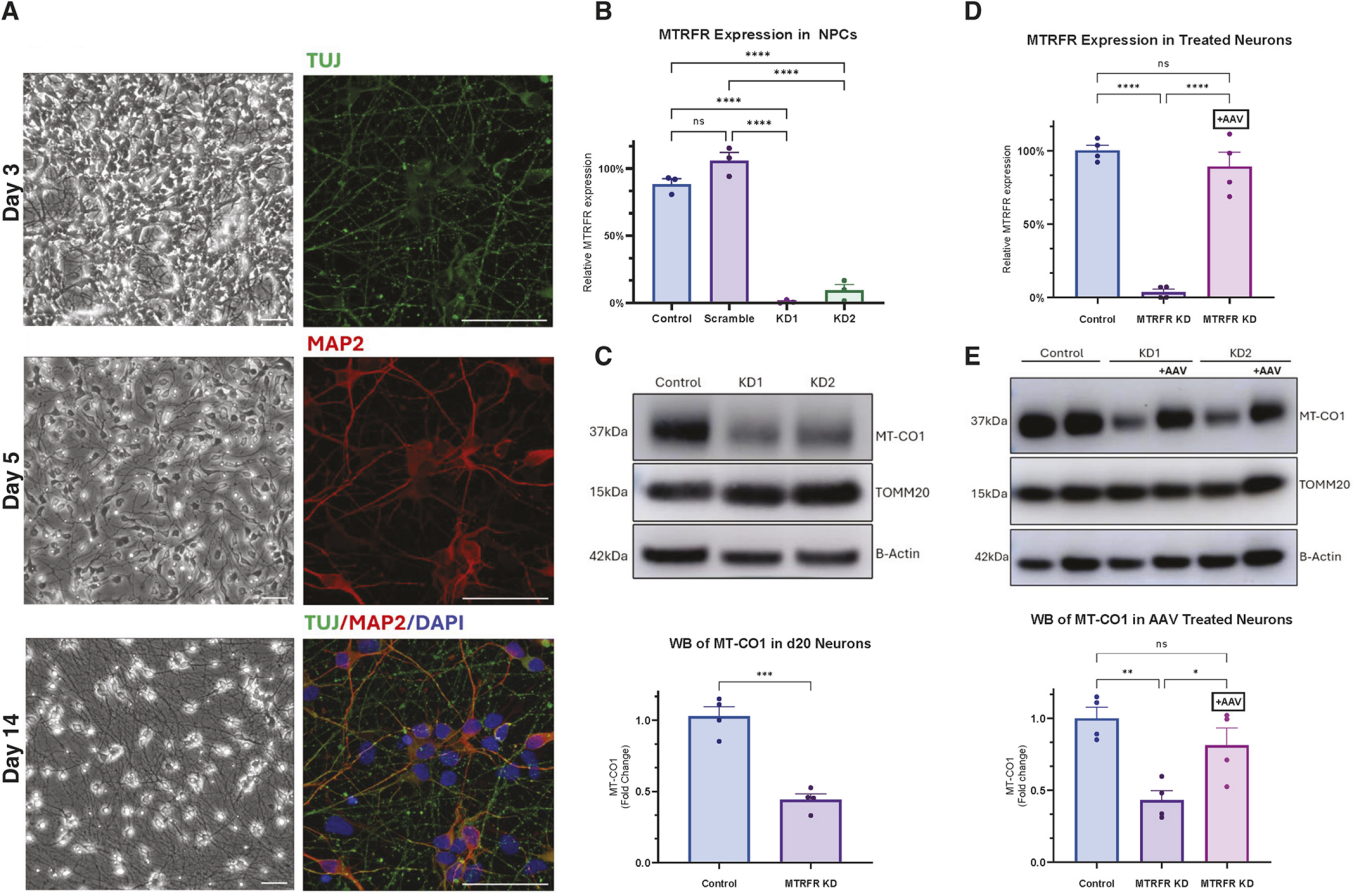
**Rescue of mitochondrial translation in an hiPSC-derived neuronal model of *MTRFR* deficiency.** (A) Representative brightfield images of NGN2-induced neuronal differentiation (left column), and immunofluorescence of the neuronal markers MAP2 and TUJ (also known as neuron-specific class III beta-tubulin) (right column). Scale bars: 25 μm. (B) Lentiviral transduction was used to express two single-guide RNAs targeting the promoter region of *MTRFR* to induce knockdown (KD) of MTRFR in human induced pluripotent stem cells (hiPSCs) constitutively expressing the dCas9-KRAB CRISPRi machinery. Successful KD of MTRFR expression was achieved with both guides based on RT-qPCR of *MTRFR* expression in neuronal progenitor cells (NPCs) using beta-actin as standard (*n*=3). A scrambled guide had no effect on MTRFR expression (*n*=3). For follow-up experiments, both KD lines were plotted together as ‘MTRFR KD’, and the scrambled line was plotted with the unedited cell line as ‘Control’. (C) KD of MTRFR in cortical neurons causes decreased mitochondrial translation efficiency 20 days after neuronal induction based on reduced MT-CO1 levels (*n*=4). Quantification was normalized to TOMM20. (D) Transduction of KD hiPSC-derived neurons with adeno-associated virus (AAV)9 expressing *MTRFR* at a multiplicity of infection of 500 restores expression levels by RT-qPCR 20 days after transduction (*n*=4). (E) AAV9 delivery of *MTRFR* restored mitochondrial translation based on western blotting and quantification of MT-CO1 levels in day 25 neurons before and after AAV9 treatment (*n*=4). Error bars are mean±s.e.m. One-way ANOVA (B,D,E) and unpaired two-tailed *t*-test (C) were used for statistical analysis. ns, not significant; *P<0.05, **P<0.01, ***P<0.001, ****P<0.0001.

Although the mouse data above indicate that lethality from loss of *Mtrfr* function can be rescued by a human transgene and that there are no adverse effects from overexpression of *MTRFR* in the mouse, the lethal phenotype of the mouse neither accurately reflects the human disease phenotype of *MTRFR* deficiency nor provides an animal model for preclinical testing. We therefore also explored a human induced pluripotent stem cell (hiPSC)-derived neuronal model of *MTRFR* deficiency generated by CRISPRi knockdown of *MTRFR*. Transcriptional repression was achieved by cloning single-guide RNAs targeting the transcription start site of *MTRFR* into a lentiviral vector followed by transduction of hiPSCs constitutively expressing the dCas9-KRAB machinery (Fig. 6A,B). The hiPSCs after downregulation of *MTRFR* were rapidly differentiated into neuronal progenitor cells (NPCs) and cortical i^3^ neurons through induced overexpression of neurogenin-2 (NGN2) (see Materials and Methods). Levels of *MTRFR* were reduced in NPCs to less than 10%, and knockdown persisted when cells were differentiated to cortical neurons (Fig. 6B). These cells had decreased levels of MT-CO1, indicating a significant decrease in mitochondrial translation (Fig. 6C). Knockdown cortical neurons were then treated with an AAV9 delivering *MTRFR* at an MOI of 500 viral genomes/cell, a dose not anticipated to cause toxic phenotypes *in vitro* (Fig. 2). Transduced neurons showed a return to normal *MTRFR* expression levels that were comparable to those of wild-type and control lines (Fig. 6D). As this CRISPRi system acts only on the genomic transcriptional start site of the endogenous *MTRFR* gene, it does not affect expression from the AAV9-delivered transgene. There was a rescue of mitochondrial translation levels, shown as a rescue of the previous MT-CO1 depletion back to control levels after AAV9 treatment (Fig. 6E). Thus, in this human cell-based disease model, restoring *MTRFR* expression by viral delivery of the wild-type gene is also effective at correcting cellular level phenotypes associated with loss of *MTRFR* activity.

## DISCUSSION

In this study, our aim was to investigate the feasibility of therapies such as virally mediated gene replacement to treat *MTRFR* deficiency. To test this, we engineered a patient-based *MTRFR* premature truncation into the mouse genome, creating a partial loss-of-function allele, and we restored gene expression using transgenic expression of the human wild-type *MTRFR* or a partially functional transgene with three C-terminal lysines changed to alanines. To our surprise, using the partially functional premature truncation did not lead to a viable mouse model in which we could mechanistically study MTRFR deficiency and disease progression but instead to one that was embryonically lethal as a homozygote. Although the mice clearly tolerate *Mtrfr* deficiency differently from humans, this model was still useful for addressing several points regarding the feasibility of an eventual gene therapy. Heterozygous mice carrying the truncation show no phenotype, indicating a cleanly recessive loss of function and no indication of a dominant negative effect of the truncated allele or haploinsufficiency. We then asked whether the lethality of homozygous mice could be rescued by the transgenic expression of human *MTRFR*, and whether there would be any harmful effects from overexpressing either the wild-type or a mutant form of *MTRFR*. The wild-type *MTRFR* transgene was able to completely rescue the lethality, even when only one copy of the transgene was present. However, because this is an ectopic transgene insertion with a synthetic promoter, this does not necessarily equate to one copy of the endogenous gene. Rescued mice were normal in all tests performed. The mutant transgene only produced one rescued mouse, and only when two copies of the transgene were present, consistent with reduced activity/efficacy. In our experimental system, there are no harmful effects of overexpressing either the wild-type of mutated human *MTRFR* transgenes in the mouse, despite evidence from *in vitro* studies that indicate that high levels of *MTRFR* overexpression in myoblasts and cultured neurons can be deleterious. Given our findings that *MTRFR* mutations are cleanly recessive and that there is no adverse effect from overexpression of *MTRFR in vivo* in mice, we conclude that this genetic deficiency is a good candidate for gene therapy approach. However, our premature truncation mouse is not a good disease model for testing the efficacy of such a strategy. In lieu of an *in vivo* model, we instead showed that restoring expression of *MTRFR* using AAV9 delivery rescues cellular phenotypes in neurons derived from a hiPSC CRISPRi knockdown model of *MTRFR* deficiency.

Based on our inability to create a viable homozygous mutant mouse for *Mtrfr*, mice are more sensitive to MTRFR deficiencies than humans, as all known patients with *MTRFR* deficiency symptoms are either homozygous or compound heterozygous for pathogenic variants in *MTRFR* including truncations as early as amino acid 84 (Antonicka et al., 2010; Buchert et al., 2013; Heidary et al., 2014; Perrone et al., 2020; Pyle et al., 2014; Shimazaki et al., 2012; Spiegel et al., 2014; Tucci et al., 2014). There is no evidence of haploinsufficiency in mice heterozygous for *Mtrfr* deficiency, which is also true in humans. Searching the Broad Institute's Gnomad database, numerous deleterious heterozygous variants are found in healthy control patients. Why mice are more sensitive to this mitochondrial disorder than humans has not yet been explored, but a mechanism conferring resilience in humans may also suggest an additional therapeutic strategy. Given the juvenile to adult onset of *MTRFR* deficiency, an embryonically lethal mouse model is not helpful for understanding disease mechanisms and progression. To generate a more valid disease model, we are currently creating a *Cre*-inducible conditional KO mouse with *LoxP* sites flanking the third exon. This model will allow us to use an assortment of *Cre*-transgenic strains to delete *Mtrfr* in specific cell types of clinical relevance, such as cells in the retina and motor neurons. Although conditional models are always artificial given that the experimenter is controlling the timing and tissue type of interest, according to the *Cre* strain used, this approach will allow us to assess cell types of interest, such as retinal neurons, that are lacking *Mtrfr* expression. As we have every reason to believe that mouse MTRFR and human MTRFR function equivalently in mitoribosome rescue, this conditional model will be able to recapitulate the disease mechanism. This, in conjunction with our work on cell-based models of *MTRFR* deficiency, including engineered hiPSC lines with a patient-associated early truncation, will allow us to uncover more about the mechanisms driving this inherited peripheral neuropathy.

When considering a gene replacement strategy, it is important to ensure that overexpression of the protein of interest is safe and to understand whether there are any dosing thresholds or restrictions. Therefore, we used transgenic mice to confirm that overexpressing wild-type *MTRFR* even in mice homozygous for the transgene and with endogenous *Mtrfr* would be safe. We found no consequences from this overexpression. Similarly, we saw no adverse effects with transgenic expression of the 3K>A mutated transgene, indicating that the presence of this mutated protein is benign. Although we did see that overexpressing *MTRFR* AAV9 at high dosages was detrimental to mitochondrial translation in cells, the level of overexpression we achieved with transgenes in these mice seems to not only be enough to rescue but also at a safe level with no harmful effects. Unfortunately, a comparison of overexpression at the RNA level was not performed in the cell culture experiments, and western blotting was unable to detect endogenous MTRFR protein without overexpression, making it difficult to compare the amount of MTRFR expression that proved deleterious in cells to the levels achieved in our transgenic mice. It has been previously reported that overexpression of some genes in a disease model as a therapy can be harmful. For example, a similar mitochondrial translation release factor mitochondrial ribosomal protein L58 (*Mrpl58*), also referred to as immature colon carcinoma transcript-1 (*Ict1*), has been linked to various types of cancers (Chicherin et al., 2021), including colorectal cancer (Lao et al., 2016), osteosarcoma (Pan et al., 2021), breast cancer (Peng et al., 2018; Wang et al., 2017a), lymphoma (Xie et al., 2017) and numerous other cancers (Chang et al., 2017; Huang et al., 2014; Li et al., 2018; Tao et al., 2017; Wang et al., 2017a,b). It has been found that MRPL58 protein content was especially elevated in larger, late-metastatic tumors (Chang et al., 2017; Pan et al., 2021; Xie et al., 2017). As MRPL58 acts as a structural component of the large subunit of the mammalian mitochondrial ribosome in addition to acting as a mitochondrial release factor on nonstop complexes like MTRFR (Richter et al., 2010), it was necessary that we confirmed that overexpression of the related *MTRFR* gene does not have the same consequences as overexpression of *MRPL58.* This was the case, as we did not find any harmful consequences of *MTRFR* overexpression, including tumor formation, in the 9-month-old *MTRFR* overexpression mice, an age at which there was time for the manifestation of any overexpression-related abnormalities. Furthermore, expression of transgenic *MTRFR* did not change endogenous *Mrpl58* levels (Fig. S4).

Gene therapy approaches are moving to the forefront of primary mitochondrial disease (PMD) research. PMDs include genetic disorders that are caused by pathogenic variants in genes that code for or are associated with the mitochondrial respiratory chain (Chinnery, 2021), such as *MTRFR* deficiency. AAV-based approaches are favored, as AAV vectors can transduce both dividing and non-dividing cells, including neurons, which are commonly affected in PMDs. For diseases like *MTRFR* deficiency, AAVs can target the multiple cell types associated with the disease and have been used in related disorders such as Leber's congenital amaurosis (LCA), dominant optic atrophy (DOA) and other mitochondrial disorders like Leigh syndrome. LCA is an inherited retinal disease, and an AAV2 therapy was approved by the US Food and Drug Administration (FDA) to treat the autosomal recessive blinding disease with success (Georgiadis et al., 2016; Le Meur et al., 2018; Simonelli et al., 2010). DOA is a form of hereditary optic neuropathy with selective degeneration of retinal ganglion cells, the axons of which make up the optic nerve. DOA is mainly caused by dominant variants in the optic atrophy like-1 (*OPA1*) gene, which encodes a large mitochondrial GTPase (Lenaers et al., 2021). Recently, mice with dominant mutations in *Opa1* showing DOA symptoms were rescued with intravitreal injections of an AAV2 carrying the human *OPA1* cDNA (Sarzi et al., 2018). An analogous approach could be feasible for treating the optic neuropathy associated with *MTRFR* deficiency; however, delivery directly to the eye would not treat the peripheral neuropathy and other symptoms of *MTRFR* deficiency. Other AAV9-associated gene therapy approaches targeting Leigh syndrome have faced challenges with organ transduction and sustainable transgene expression; however, these studies are encouraging for further treatment approaches (Di Meo et al., 2017; Lake et al., 2016; Ling et al., 2021). A primary challenge of clinical translation of gene therapy treatments is safe dosing in patients while also getting adequate transduction and expression in various organs. Like Leigh syndrome and DOA, *MTRFR* deficiencies are a good candidate for gene therapy treatment, and our studies have shown that we are able to get adequate expression in various organs without adverse effects of the *MTRFR* transgenes. The rescue of mitochondrial translation in hiPSC-derived cortical neurons further indicates that restoring expression of MTRFR restores function in relevant human cell types.

Neuropathy resulting from mitochondrial dysfunction is not uncommon and, interestingly, most neuropathies associated with mitochondrial diseases are primarily axonal (Luigetti et al., 2016). In both adults and children, peripheral neuropathy can be the main clinical presentation of some primary mitochondrial diseases (Horvath et al., 2023). Although mitochondrial dysfunction plays an integral role in CMT, Leigh syndrome and Behr's syndrome, there are currently no treatments for these inherited peripheral neuropathies (Horvath et al., 2023). Therefore, it is essential to determine how dysfunctional mitochondrial translation caused by *MTRFR* deficiency leads to neurodegeneration to identify potential therapeutic targets. Additional work is needed to discover potential therapeutic targets and treatment options. It is known that both peripheral and optic nerves are very susceptible and sensitive to energy failure, but this is observed only in some mitochondrial disorders (Luigetti et al., 2016), such as *MTRFR* deficiency. It is estimated that a third of patients with mitochondrial diseases have a neuropathy, whether their mitochondrial disease is due to pathogenic variations in the nuclear DNA (nDNA) or mtDNA (Bindu et al., 2016; Pareyson et al., 2013). In summary, mitochondrial diseases are among the most clinically heterogeneous and debilitating class of diseases. To develop a treatment for patients living with *MTRFR*-related deficiencies, understanding the potential harmful consequences of overexpression of MTRFR, whether the presence of a truncated protein causes adverse effects and whether we can rescue severe phenotypes with a genetic overexpression are all key questions that are answered in this study. There is still much work to be done to determine whether there are adverse effects to MTRFR overexpression in various systems, determining the tissues and cell types that need to be targeted, to see whether inducing expression later in life or heterogeneously in different tissues and cell types could have detrimental effects, including immunogenicity against the therapeutic protein in patients or animals that are null for the endogenous allele, and to establish whether treatment post disease onset works. Eventually therapeutics that elevate *MTRFR* expression, by an AAV-delivered gene or another approach, will need to be thoroughly tested and pass rigorous safety and toxicology studies. We observed a full rescue of MTRFR-deficient mice with transgenic expression of a human *MTRFR* gene with no adverse effects, and we rescued cortical neurons *in vitro* with AAV9 treatment. Our goal with this paper was to provide proof-of-concept analysis and provide a first step towards developing a therapeutic for *MTRFR* deficiencies. More work is needed to uncover the exact mechanism behind this disease, but these findings are an encouraging step forward to potentially treating *MTRFR* deficiencies with gene therapies.

## MATERIALS AND METHODS

### Protein sequence alignment of human and mouse MTRFR

Protein sequences for the human and mouse MTRFR were taken from UniProt and aligned using T-Coffee and Espresso multiple sequence alignment tool through the Centre for Genomic Regulation of Barcelona (Armougom et al., 2006; Di Tommaso et al., 2011; Notredame et al., 2000; O'Sullivan et al., 2004; Poirot et al., 2004). The MTS was based on UniProt prediction of the human protein sequence's MTS.

### MEF generation and culturing

MEFs with KO of *Mtrfr* were generated by mating heterozygous C57BL/6-A^tm1Brd^ Mtrfr^tm1(KOMP)Mbp^/Oulu males and females from the Infrafrontier/European Mouse Mutant Archive resource, strain EM:09863. Mice were maintained at the University of Helsinki animal facility under license ESAVI/3043/04.10.07/2016. MEFs were immortalized by retroviral transduction of E7 and hTERT (Lochmuller et al., 1999), and cultured in Dulbecco's modified Eagle's medium (DMEM) with high glucose (Sigma-Aldrich, D6429) supplemented with 10% fetal bovine serum (Gibco, A5256701), 1× glutamax (Gibco, 35050-038) and 50 μg/ml uridine (Sigma-Aldrich, U3003), which acts to bypass deficits in pyrimidine biosynthesis that occur with mitochondrial dysfunction. Cells were grown at 37°C and 5% CO_2_.

### Human myoblast generation and culturing

Wild-type human myoblasts were grown in Skeletal Muscle Cell Growth Medium (Sigma-Aldrich, 151-500) supplemented with uridine 50 μg/ml (Sigma-Aldrich, U3003) at 37°C and 5% CO_2_. Human myoblasts were a gift from Dr Eric Shoubridge at McGill University and have been previously described (Boulet et al., 1992).

### Primary embryonic cortical neuron cultures

Primary embryonic cortical neuron cultures were derived from pregnant wild-type NMRI mice (obtained from Charles River Laboratories and kept at the University of Helsinki animal facility under license KEK21-012). Pregnant NMRI mice were sacrificed via CO_2_ and cervical dislocation, hysterectomies were performed to collect E15-E17 mouse embryos. Cortices from embryos were dissected as previously described (Changou et al., 2017), then pooled to isolate primary neurons. Briefly, dissected cortices were collected in a standard 15 ml canonical tube, washed thrice with Hank's balanced salt solution (HBSS) without Ca^2+^ or Mg^2+^ (Gibco, 14175-053), incubated on ice for 10 min and semi-digested with trypsin (MP Biomedicals, 103139) for 15 min at 37°C. To isolate individual cells, trituration was done once with HBSS with 10% fetal bovine serum (FBS; Gibco, 10500056) and DNAseI (Roche, 11284932001), then twice with only HBSS with 10% FBS. Cells were centrifuged at 100 ***g*** for 1 min, resuspended, and centrifuged again at 25 ***g*** for 30 s in culture medium. Supernatant was collected in a new canonical tube, centrifuged at 100 ***g*** for 2 min for the final washing step, then resuspended in medium. Isolated cells were maintained in neurobasal medium (Gibco, 21103049), containing 1×B27 Supplement (Gibco, 17504-044), 1× Glutamax (Gibco, 35050-038) and 100 µg/ml Primocin (Invitrogen, ant-pm). Cells were plated on pre-coated (0.01% poly-L-lysine; Bio-Techne Cultrex, 3438-100-01), six-well (1-2×10^6^ cells/well) or 10 cm (14.6×10^6^ cells/plate) plates, then kept at 5% CO_2_, 37°C. Half-medium changes were done every 3-4 days until sample collection.

### Retrovirus treatment of MEFs

The cDNAs of wild-type mouse *Mtrfr*, human *MTRFR*, the GSQ mutant (in the GGQ catalytic domain), the C-terminal deletion series (A134, K141, K151) and *MTRFR* with the point mutations in the C-terminal domain (K146A, K153A, K156A) were Gateway cloned into converted pBABE-puro or pMXs-IRES-Blasticidin retroviral vectors. All cloning was verified by Sanger sequencing. Retroviruses were generated using the Phoenix amphotropic packaging line (Ng et al., 2022). Transduced cells were used directly in experiments following selection with puromycin or blasticidin. In these experiments, the individual viruses were generated at the same time, side by side, with the same packaging line, and transduced into recipient cells at the same time to ensure that these experiments were controlled. These experiments were also repeated multiple independent times to ensure reproducibility of the transduction event. Following retroviral transduction via application of the retroviral vectors to the cell culture medium, recipient cells were put under antibiotic selection for 3 days with puromycin and/or blasticidin. This standard selection method for retroviral transduction will kill all non-transduced cells (Swift et al., 2001). Transduction methods can also be found in previous co-author publications (Ng et al., 2022; Richter et al., 2018, 2015, 2019).

### AAV treatment of myoblasts and cortical neurons

The wild-type human *MTRFR*, the GSQ catalytic mutant and the C-terminal deletion at residue A134* were cloned into the AAV9 vector pSubCAG-WRPE by PCR using KAPA HiFi into the PmlI and MluI restrictions sites. A134* was used here as a control owing to the stability of the mutant protein with expression levels that are very similar to the GSQ mutant. This ensures that the reduced MT-CO1 phenotype with the GSQ mutant was specific to the GSQ variant and not due to non-specific toxicity from mitochondrial-targeted overexpression of exogenous proteins such as MTS-eGFP. All cloning was verified by Sanger sequencing. AAV9s were produced by the AAV Gene Transfer and Cell Therapy core facility at the University of Helsinki. Mouse embryonic cortical neurons were plated onto six-well plates pre-coated with poly-L-lysine at a density of 1×10^6^ cells/well. AAV9 vectors were administered with a half-medium change at 2 days *in vitro* (DIV2) at an MOI of 2×10^2^, 2×10^3^, 2×10^4^ viral genomes/cell. Half of the medium was changed every 3-4 days until cells were harvested on DIV20.

### Immunoblotting of MEFs, myoblasts and mouse embryonic cortical neurons

Whole-cell lysates were prepared using phosphate-buffered saline, 1% n-dodecyl-β-D-maltoside (Thermo Fisher Scientific, 329370010), 1 mM phenylmethylsulfonyl fluoride (Sigma-Aldrich, 93482) and protease inhibitor (Thermo Fisher Scientific, A32955). Protein concentrations were measured by the Bradford assay (Bio-Rad, 500-0006). Equal amounts of proteins (20 μg) were separated by 12% Tris-glycine sodium dodecyl-sulfate–polyacrylamide gel electrophoresis (SDS-PAGE) and transferred to nitrocellulose membrane by semi-dry transfer. Membranes were blocked with 1.5% milk in Tris-buffered saline with 0.1% Tween 20 (Sigma-Aldrich, P1379) (TBST) for 1 h. This was followed by overnight incubation with the primary antibodies at 4°C in 5% bovine serum albumin/TBST and detected the following day with secondary horseradish peroxidase (HRP)-conjugated antibodies (Goat Anti-Rabbit IgG HRP conjugate, Jackson ImmunoResearch, 111-035-144 and Goat Anti-Mouse IgG HRP conjugate, Jackson ImmunoResearch, 115-035-146) using ECL with Azure 300 (Azure Biosystems) or iBright imager (Invitrogen). Primary antibodies were as follows: anti-uL11m (Proteintech, 15543-1-AP; 1:20,000), anti-mS26 (Proteintech, 15989-1-AP; 1:5000), anti-mL45 (Proteintech, 15682-1-AP; 1:10,000), anti-MT-ATP6 (Proteintech, 55313-1-AP; 1:2000), anti-MT-CO1 (Abcam, ab14705; 1:10000), anti-SDHA (Abcam, ab14715; 1:10,000), anti-calnexin (Proteintech, 10427-2-AP; 1:2000) and anti-GFP (ChromoTek, 3H9; 1:5000). An affinity purified rabbit polyclonal antibody was raised against MTRFR using peptide C-EQFVKGHGPGGQAT (residues 62-75), which is common to both human and mouse proteins, by BioGenes GmbH (Germany). Here, ‘C-’ denotes an artificial N-terminal cysteine that can be used for conjugation of the peptide to carrier proteins for immunization. The antibody was generated at BioGenes GmbH. Note that owing to the low expression levels of MTRFR, we were unable to detect MTRFR by western blotting without overexpression in cell culture, and we were still unable to detect the protein in mitochondrial fractions in our *in vivo* models, even with transgenic overexpression.

### Radioisotope labeling of mitochondrial translation

A 30-min pulse labeling of mitochondrial nascent chains with ^35^S Met-Cys (EasyTag, Perkin Elmer) in cultured MEFs was performed as described (Richter et al., 2013). *Mtrfr*-KO MEFs were treated with either an empty vector, wild-type MTRFR or MTRFR with premature truncations at either amino acid E160, K151, R141 or A134. All samples were treated with Benzonase (Sigma-Aldrich) according to the manufacturer's instructions and then mixed with gel loading buffer (186 mM Tris-HCl pH 6.7, 15% glycerol, 2% SDS, 0.5 mg/ml Bromophenol Blue and 6% β-mercaptoethanol). A 12-20% gradient SDS-PAGE was used to separate samples, which were then dried for exposure with a Phosphoscreen and scanned with a Typhoon 9400 (GE Healthcare). Gels were rehydrated in water and Coomassie Blue stained to confirm loading.

### Maintenance of the *Mtrfr*^K155*^ and transgenic mouse strains

All procedures were performed according to the guidelines of the National Institutes of Health Guide for the Care and Use of Laboratory Animals and were approved by the Institutional Animal Care and Use Committees of The Jackson Laboratory. All mice were bred and housed in the research animal facility at The Jackson Laboratory. Mice were maintained in a medium barrier pathogen-free room and housed in pressurized intraventilated caging with a 14 h:10 h light–dark cycle. Mice were provided acidified water and 6% fat pelletized chow *ad libitum*.

### Generation of K155* line

All genetically altered mice were generated by the Genome Engineering Technology service at The Jackson Laboratory on a C57BL/6J background. Stop codons were inserted at amino acids 155 and 156 in the endogenous *Mtrfr* gene using CRISPR/Cas9 genome editing to induce a premature truncation in the alpha-helix C-terminus of the protein (AAA AAA codons changed to TAA TAA). Two guides targeting exon 1 of the *Mtrfr* gene and spaced ∼80 bp apart were used to create double-strand breaks. Guide sequences are upstream (GATCCACAGATCTTGTTTGG) and downstream (GATGCTGATGGTATACCAAG). The two stop codons at 155 and 156 were introduced using a plasmid as a template for repair by homologous recombination. The plasmid included a 1.2 kb region of homology upstream of the first guide and an 850 bp region of homology downstream of the second guide. Potential founders were identified by Sanger sequencing of genomic PCR products and were bred at least two generations to wild-type C57BL/6J mice to eliminate mosaicism and possible off-target effects. Integration at the correct genomic locus was confirmed by sequencing long PCR products generated using primers outside the regions of homology (F, 5′-AAAGGGTGGATTGAGGGACT-3′; R, 5′-CAGCAATCCTCCTGCCTTAG-3′) followed by Sanger sequencing of the PCR products to confirm heterozygosity for the introduced mutations. These mice were genotyped using Sanger sequencing of genomic DNA PCR amplification products with the following primers: K155* F, 5′-AAAGGGTGGATTGAGGGACT-3′; K155* R, 5′-CAGCAATCCTCCTGCCTTAG-3′.

### Generation of wild-type and 3K>A transgenic *MTRFR* lines

The Genome Engineering Technology core at The Jackson Laboratory also generated transgenic lines on a C57BL/6J background, one containing a wild-type form of the human *MTRFR* gene, the other carrying a mutated form of the human *MTRFR* gene with three C-terminal lysines changed to alanines (K146, 153, 156, referred to as 3K>A) to create a predicted partial loss of function. These transgenes were inserted into the *Rosa26* safe harbor locus on chromosome 6 using CRISPR/*Cas9* genome engineering. Both transgenes were engineered using the same strategy (sequences are provided in Fig. S2). A guide RNA (ACTGGAGTTGCAGATCACGA) was used to introduce a double-strand break in the *Rosa26* locus. A double-stranded plasmid was used as the template for repair by homologous recombination, with a 1.96 kb region of homology upstream of the *Rosa26* insertion site and a 2.53 kb region of homology downstream of the insertion site. The inserted sequence consisted of the CAG/CMV enhancer and beta-actin promoter upstream of an intron (beta-actin intron1), a LoxP-flanked stop cassette consisting of three SV40 poly-adenylation signals, the MTRFR open reading frame (wild-type or 3K>A) and a growth hormone 3′ untranslated region and poly-adenylation sequence. Correct genomic integration was confirmed by using PCR primers outside each homology arm, amplifying to insertion-specific sequences (5′ LR-PCR F, 5′-GAGTCCAAGAATGTGAGGTGG-3′ and R, 5′-CCCCCCAGAATAGAATGACA-3′; 3′ LR-PCR F, 5′-TATGGTAATCGTGCGAGAGG-3′ and R, 5′-AGAATCTGACCTGCAAGTTCC-3′). Once founders were identified, they were bred at least two generations to wild-type C57BL/6J mice to eliminate mosaicism and possible off-target mutations. Although these mice were generated as conditional transgenics with a LoxP-flanked stop cassette preventing expression in the absence of *Cre*, constitutively expressing substrains were established by breeding to a ubiquitously expressed *Cre* to remove the stop cassette in the germline. Founders of these substrains that carried a deletion of the stop cassette, but not the *Cre* transgene, were used to establish the constitutively expressing transgenic lines that were used in these studies. The lines are referred to as 3K>A-Tg and WTKI-Tg. These mice were genotyped using standard PCR with the following primers: *MTRFR*-Tg F, 5′-TATGGTAATCGTGCGAGAGG-3′; *MTRFR*-Tg R, 5′-CCCCCCAGAATAGAATGACA-3′.

### K155* embryo collection and chi-squared analysis

K155* heterozygous mice were used in timed matings, and embryonic harvests were performed at varying gestational timepoints: E10.5, E11.5, E12.5, E13.5 and E14.5. Mice were mated, and the males were removed after 24 h. Females were monitored until desired timepoints were reached, then euthanized with CO_2_, and embryos were dissected out. The recovered embryos were washed in 1×PBS briefly and then fixed in 4% PFA before paraffin embedding, sectioning and H&E staining. A portion of the tail was taken from each embryo for genotyping. Chi-squared analysis was performed using the equation *x*^2^=Σ((*O*_i_−*E*_i_)^2^/*E*_i_), where *O*_i_ is the observed value and *E*_i_ is the expected value; degree of freedom was equal to 2. The equation was carried out using K155* embryo genotypes to showcase a statistical reduction in genotype presence compared to expected genotype ratios.

### Body weights and muscle weight to body weight ratio

Body weight was assessed longitudinally as indicated in figures, separated by sex and analyzed using one-way ANOVA. To test for selective muscle atrophy, the triceps surae muscles (medial and lateral gastrocnemius, soleus and plantaris) were dissected free and weighed. Final body weights and triceps surae weights were used to calculate muscle weight to body weight ratios, which did not show sex effects and were plotted as pooled data.

### Analysis of mRNA expression using RT-qPCR

Total RNA was extracted from snap-frozen liver or spinal cord samples via Trizol (Thermo Fisher Scientific, 15596026) homogenization. Chloroform was then added, the aqueous layer was separated, 1.5 volumes of ethanol were added, and the solution was transferred to an RNeasy Mini column (Qiagen, 74104). The rest of the kit protocol for RNA purification with on-column DNase I digestion (Qiagen, 79254) was followed. The purified RNA was reverse transcribed using standard techniques (Thermo Fisher Scientific, 18080400), including a control that did not contain any reverse transcriptase. cDNA was used for standard quantitative PCR using SYBR green reagents (Thermo Fisher Scientific, A46109) and run on a ViiA7 machine (Thermo Fisher Scientific). Primers for RT-qPCR and standard PCR for *Mtrfr* were as follows: *Mtrfr* F, 5′-CTTCAGGAGAAGCCAGCACT-3′; *Mtrfr* R, 5′-CCCGTCTCTACCTTGACCAC-3′. Primers for RT-PCR and Sanger sequencing for *MTRFR* were as follows: *MTRFR* F, 5′-GCAGTTTGTGAAAGGACACG-3′; *MTRFR* R, 5′-CCGCTTCTCG-TTTTTCTTTG-3′. Primers for additional qPCR target genes *Mrpl58*, *Fgf21*, *Gdf15* and beta-actin were as follows: *Mrpl58* F, 5′-TGAGCTGTCACTGGACTTGG-3′; *Mrpl58* R, 5′-TGACCTGGGGAACTGCTAAC-3′; *Fgf21* F, 5′-CTGCTGGGGGTCTACCAA-3′; *Fgf21* R, 5′-CTG-CGCCTACCACTGTTC-3′; *Gdf15* F, 5′-GGGACCCCAATCTCACCT-3′; *Gdf15* R, 5′-GAGCTACGGGGTCGCTTC-3′; beta-actin F, 5′-GATCTGGCACCACACCTTCT-3′; beta-actin R, 5′-GGGGTGTTGAAGGTCTCAAA-3′. Cycle threshold (Ct) values as determined by the software (QuantStudio, Thermo Fisher Scientific) were used to calculate arbitrary expression units, which are equal to 100 times the fold change. Delta-delta Ct (ΔΔCt) values were calculated using beta-actin as the reference gene and the average of all wild-type samples as the reference group. *P*-values were determined in Prism (GraphPad) using one-way ANOVA. Methods for assessing ATF4-target gene expression (*Gdf15*, *Fgf21*) have been previously reported (Hines et al., 2023; Spaulding et al., 2021, 2016).

### Tissue preparation for histology and electron microscopy

Mice were euthanized using CO_2_, and heart, liver, kidney, spleen, brain and a portion of spinal cord were harvested and fixed in Bouin’s fixative. Medial gastrocnemius muscles were weighed before fixation in Bouin’s fixative and used to calculate muscle weight to body weight ratios. Whole eyes were dissected out and fixed in 4% PFA. Tissues were sent to the Histology service at The Jackson Laboratory for embedding, sectioning and H&E staining. A portion of spinal cord was fixed using 10% neutral buffered formalin (Fisher Scientific, 22-050-104), embedded, sectioned and stained for ubiquitin. All sections were then analyzed by a veterinary pathologist (D.C.) for possible abnormalities. Sciatic and motor and sensory branches of the femoral nerves were dissected free, and fixed in 2% PFA, 2% glutaraldehyde in 0.1 M cacodylate buffer for 12 h at 4°C. The nerves were then rinsed in PBS three times for 10 min and allowed to sit in 1% osmium tetroxide (OsO_4_) in H_2_O for 2 h at room temperature (RT). The samples then underwent a dehydration gradient and soak in 40%, 60%, 80% and 95% ethanol for 15 min each at RT before a final dehydration step of a 3-min soak in 100% ethanol at RT. After this, the nerves were allowed to rest in propylene oxide for two 10-min periods at RT and were then infiltrated starting with a 1:1 ratio of propylene oxide:Embed 812 resin [7 g N-methylaniline, 13 g Embed 812 resin (Electron Microscopy Sciences, 14120), 8 g dodecenylsuccinic anhydride, 0.6 g DMP30] overnight, switched to a 1:3 ratio of the same reagents mentioned above for 4 h, before ending in 100% Embed 812 resin overnight. The nerves in 100% resin were then placed in molds and cured at 65°C for 24 h. Finally, 1 μm-thick sections were collected on slides and stained with Toluidine Blue. Both optic nerves were taken from mice, fixed, embedded and sectioned as above, and stained with either Toluidine Blue or PPD to visualize myelin and axon damage, respectively. These slides were imaged on a Nikon Eclipse 600 microscope with DIC-Nomarski optics with a 40× objective. Following the same fixation, infiltration and embedding protocol mentioned above, optic nerves were cut into ultrathin sections (90 nm) using a Leica EM Uc7 ultramicrotome (Leica Microsystems) with a diamond knife, then placed onto 300 mesh copper grids and stained using 2% uranyl acetate and Reynolds lead citrate. Samples were then evaluated at 80 kV using a JEOL JEM-1230 transmission electron microscope, and images were collected with an AMT Nanosprint15 MK-11 sCMOS digital camera at 4000×.

Tissues were examined for signs of pathology, including possible abnormal, thin or missing myelination, degenerating and/or regenerating axons, and abnormalities in the axoplasm. Axon number, axon area and nerve area were measured using an automated method in Fiji/ImageJ that was manually confirmed by visual inspection and compared between genotypes for optic and femoral nerves. Images taken of either the optic or femoral nerves were stitched together using Adobe's Photoshop photomerge feature and opened in Fiji/ImageJ. Stitched images were then converted to grayscale, flattened and converted on an 8-bit image. The scale was set depending on the pixel size of the camera. Using the threshold function, thresholds were set so that only myelin around the axons was visible, and, using the analyze particles function where size=1-Infinity and Circularity=0.5-1.0, the program generated regions of interest (ROIs). These ROIs were then manually assessed and curated to ensure that all of the selected areas were within the axon and myelin and that no axons were missed. Any axons initially missed were added manually using the wand tool with the tolerance set to 30 in legacy mode; similarly, anything that was not an axon that was selected as a ROI was removed. The data produced from this inform on the number of axons within the nerve, the axon area and the size distribution of axons within the nerves.

### Inverted wire hang test

To test muscle endurance and grip strength, we used the inverted wire hang test (Hines et al., 2023; Spaulding et al., 2016). In this assay, mice were placed on a wire grid, which was then inverted for a three 1-min trials, and the latency for the mice to fall was measured. The latency time was averaged for the three trials per testing session, and those data were used. Ages and genotypes are noted in the figures and legends. Mice were not trained prior to the initial test.

### Electromyography

Mice were anesthetized with 2% isoflurane and placed on a thermostatically regulated heating pad set to 37°C to maintain body temperature throughout the procedure. Three recording electrodes were place subcutaneously as ground and (+) in the plantar region of the left and right hindpaw, respectively, and (−) between the last two digits of the right hindpaw. Nerves were stimulated distally at the ankle, then proximally at the sciatic notch. Compound muscle action potential amplitude (CMAP), the summation of action potentials from flexor digitorum brevis muscles in the plantar hindpaw, and latency to CMAP peak from each stimulus were recorded, and the distance between the points of stimulation was measured using calipers. NCV was calculated using the following equation: [conduction distance/(proximal latency−distal latency)] (Hines et al., 2023).

### Retinal phenotyping

Retinal images were taken using a 40× objective on Nikon Eclipse 600 microscope with DIC-Nomarski optics from slides with whole-eye sections. Images were taken adjacent to the optic nerve head for consistency. The number of cells in 100 µm of the RGC layer was counted to look for signs of possible RGC loss. Retinal thickness was determined by measuring from the bottom of the photoreceptor layer to the bottom of the RGC layer.

### Generation of *MTRFR*-knockdown hiPSC line

Single-guide RNAs targeting the transcription start site of *MTRFR* were designed using CRISPick from the Broad Institute. Both the target sequence and a scrambled control sequence were then cloned into a lentiviral vector as previously described (Sanjana et al., 2014). The LentiGuide-puro pKLV-U6gRNA(BbsI)-PGKpuro2ABFP was Addgene plasmid #50946 (RRID:Addgene_50946). For lentivirus production, HEK293T cells were co-transfected with the generated transfer plasmids and the packaging plasmids pVSVg (Addgene plasmid #8454) and psPAX2 (Addgene plasmid #12260). hiPSCs with an integrated CRISPRi machinery were a gift from Dr Evan Reid (University of Cambridge, Cambridge, UK). The hiPSCs, which stably express the dCas9-KRAB transcriptional repression system, were transduced with the packaged lentivirus overnight. After 24 h, puromycin selection was performed, and the cells were left to recover. *MTRFR* gene expression levels were measured by real-time quantitative PCR. The guide RNA that achieved the most-efficient knockdown was used in subsequent AAV9 experiments.

### Transcription factor-mediated differentiation of hiPSCs into cortical neurons and AAV9 treatment

Prior to differentiation, hiPSCs were cultured in Essential 8 medium (Thermo Fisher Scientific) and passaged with ReLeSR (STEMCELL Technologies). Differentiation into cortical neurons was carried out following the protocol described (Fernandopulle et al., 2018). In short, the hiPSCs had a *NGN2* transgene stably integrated under a doxycycline-inducible promoter. At ∼80% confluency, hiPSCs were dissociated into single cells with Accutase (Corning), replated into Geltrex (Gibco)-coated plates and cultured in neuronal induction medium [DMEM-F12 (Gibco), N2 (Gibco), doxycycline (Thermo Fisher Scientific)]. Exposure to doxycycline induces the overexpression of NGN2, which in turn leads to the rapid differentiation of the hiPSCs into cortical neurons. Media changes with fresh doxycycline were performed daily. On day 4 of induction, NPCs were dissociated into single cells and replated on polyethyleneimine (MilliporeSigma)- and Geltrex-coated plates. From this point onwards, cells were cultured in neuronal maturation medium [Brainphys (STEMCELL Technologies), NeuroCult SM1 (STEMCELL Technologies), BDNF (Bio-Techne), NT3 (PeproTech), penicillin–streptomycin (Thermo Fisher Scientific)], and media changes were performed every 3-4 days until collection at day 25. For gene therapy experiments, cells were transfected at day 5 with the wild-type *MTRFR* AAV9 vector at an MOI of 500 viral genomes/cell. This dose was chosen based on previous *in vitro* experiments (Fig. 3) in which no adverse effects were seen at a dose of 10^2^ MOI treatment, but we did see minor effects from 10^3^ MOI. Thus, we chose an intermediate dose (5×10^2^) to maximize the level of overexpression while also reducing the harmful effects of overexpression. Cells were cultured with the virus for ∼24 h, after which regular media changes were performed as previously described. One-way ANOVA tests were used for statistical analysis.

### Statistics

Statistics used for each experiment are noted in the figure captions along with the genotype and cohort size for each group. For chi-squared analysis, two degrees of freedom were used for each test. Statistical analysis between more than two groups were done using one-way ANOVA. Unpaired two-tailed *t*-tests were used for pairwise comparison. Kolmogorov–Smirnov and Mann–Whitney tests were used when comparing axon area sizes of the optic nerve. *P*<0.05 was considered significant.

## Supplementary Material

10.1242/dmm.052120_sup1 Supplementary information
