# Supplementary material for: Evaluating the feasibility of gene replacement strategies to treat MTRFR deficiency
Source: Dis Model Mech. 2025 Jun 2;18(5):dmm052120. doi: 10.1242/dmm.052120 (PMC12171093; doi:10.1242/dmm.052120)
Supplement: Supplementary information [file dmm-18-052120-s1.pdf]

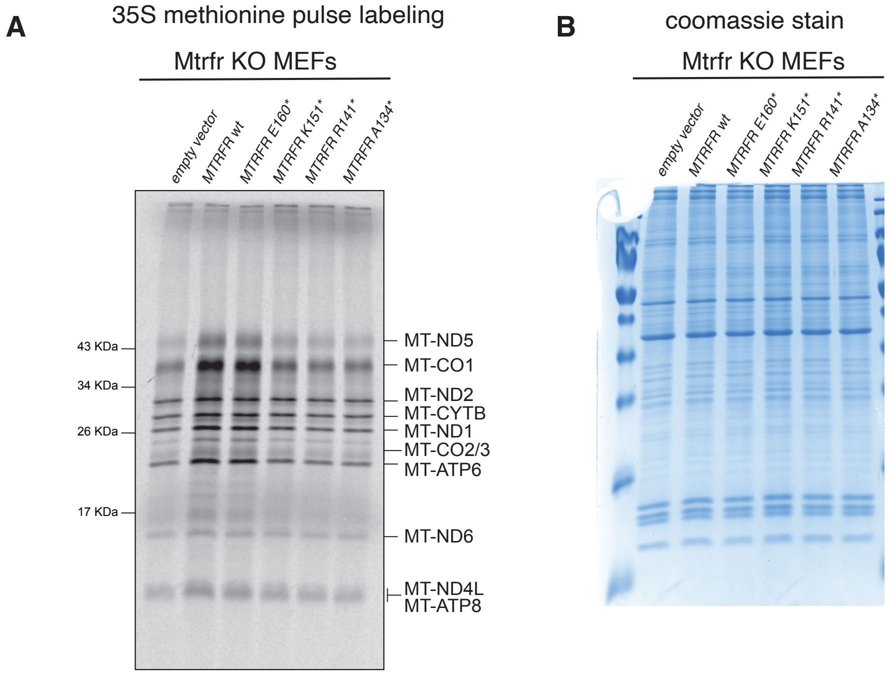

Fig. S1. Resuce of mitochondrial translation in KO-*Mtrfr* mouse embryonic fibroblasts with truncating *MTRFR* cDNA constructs. A) ^{35}S methionine pulse labeling protein blot of whole cell lysates of KO-*Mtrfr* mouse embryonic fibroblasts (MEFs) following retroviral transduction with human wildtype or prematurely truncated MTRFR cDNA. MTRFR Constructs were prematurely truncated at amino acids 160, 151, 141 and 134. Blot depicts newly synthesized proteins synthesized by the mitochondria (MT-ND5, MT-CO1, MT-ND2, MT-CYTB, MT-ND1, MT-CO2/3, MT-ATP6, MT-DN6, MT-ND4L and MT-ATP8), and that the level of rescue of mitochondrial translation is dependent on the severity of the truncation in the MTRFR protein is. B) Coomassie staining of 35S methionine pulse labeling blot to ensure there was consistent loading of the blots.

Knock in of the *CAG-L-S-L*-C12ORF65-WT cassette into *ROSA26* locus

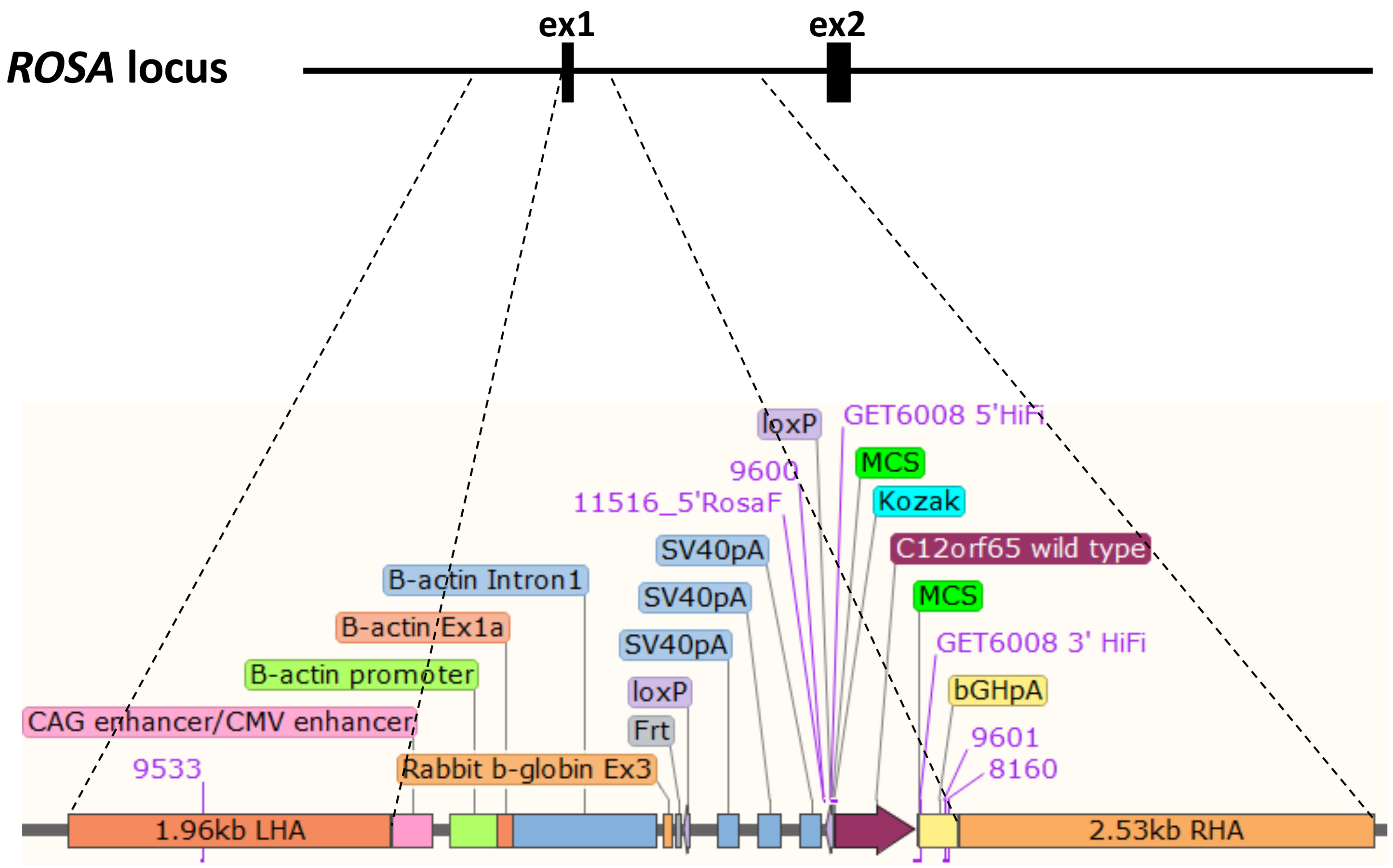

Disease Models & Mechanisms • Supplementary information

Rosa26 KI Allele

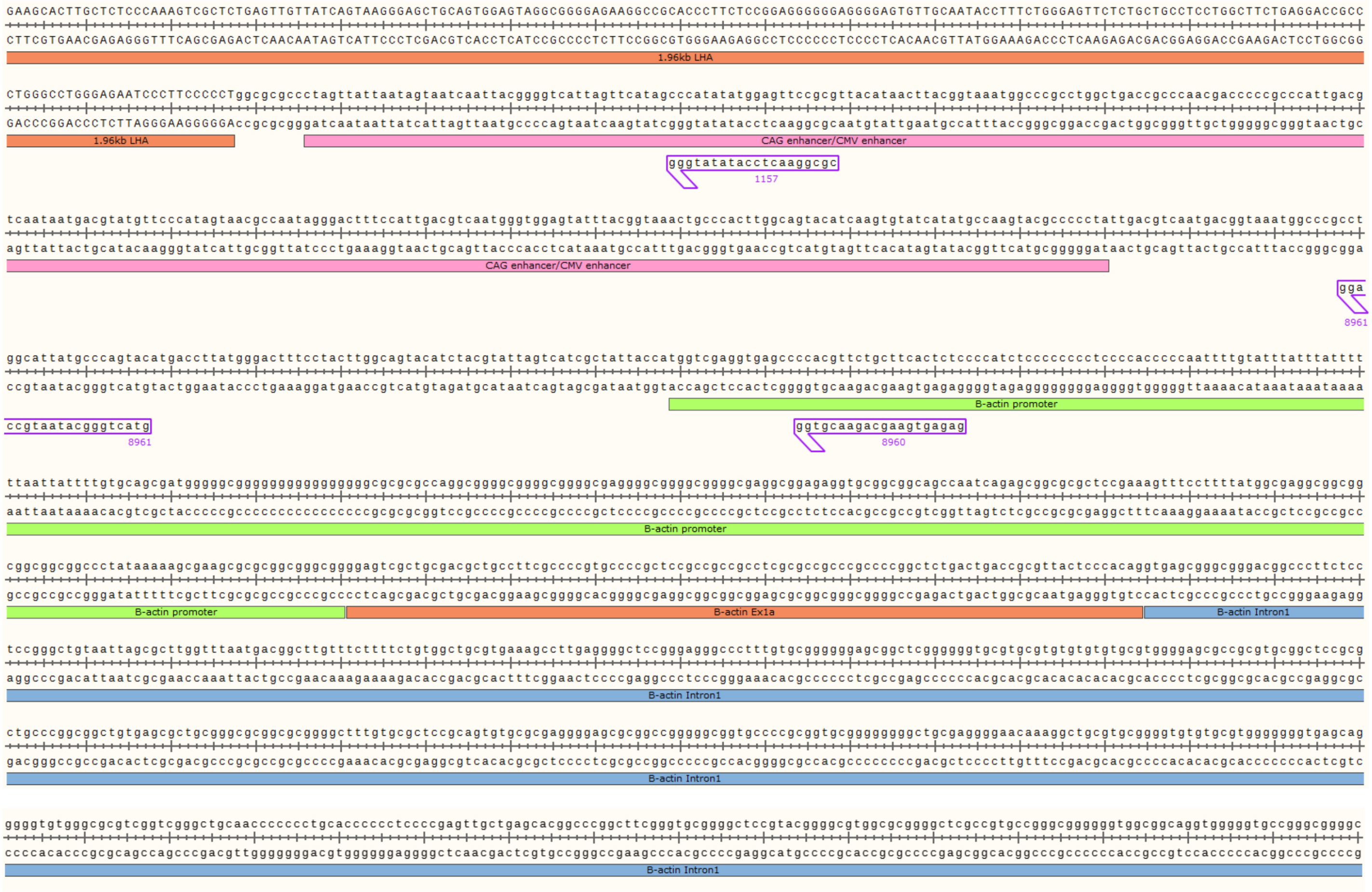

Disease Models & Mechanisms • Supplementary information

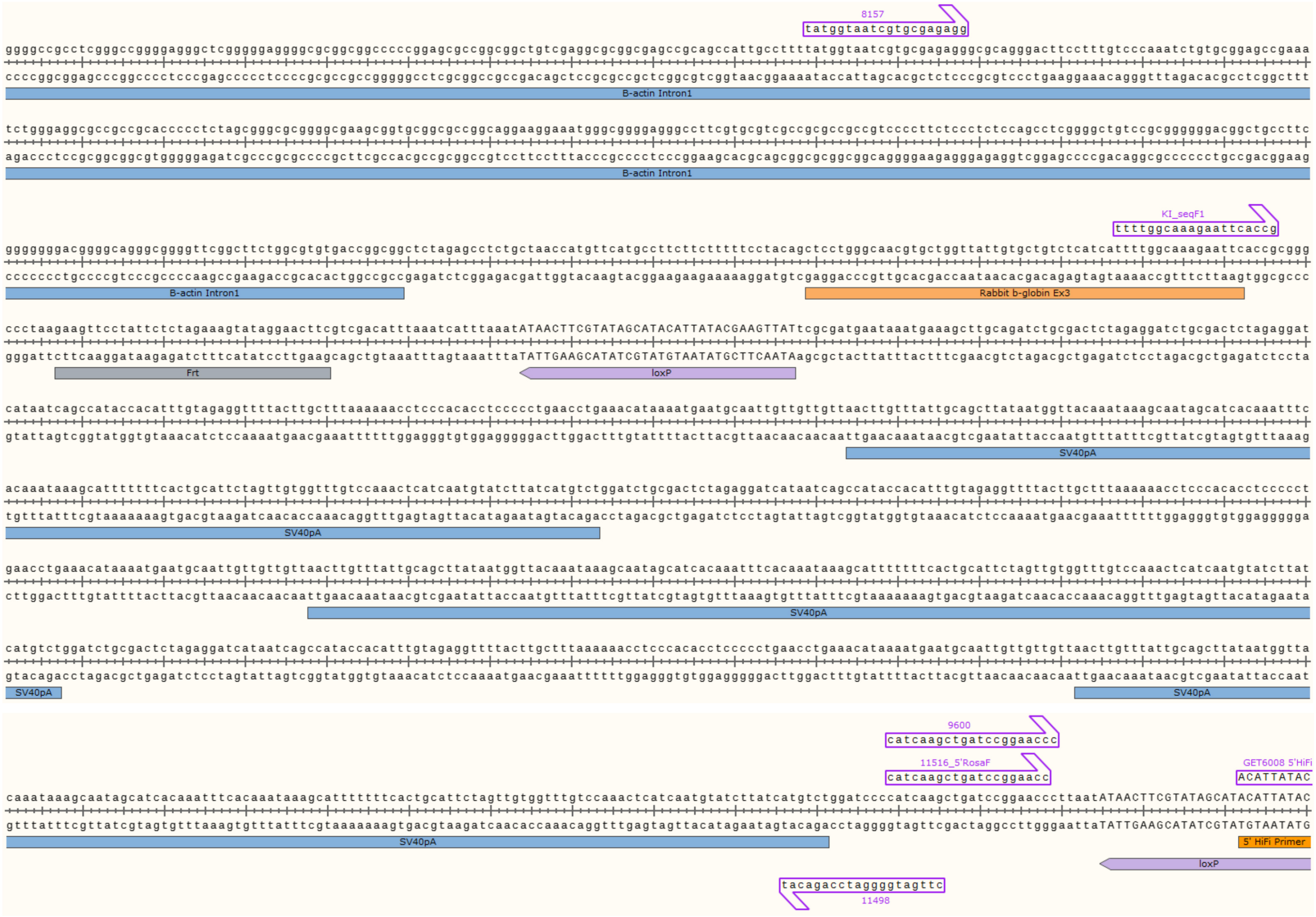

Disease Models & Mechanisms • Supplementary information

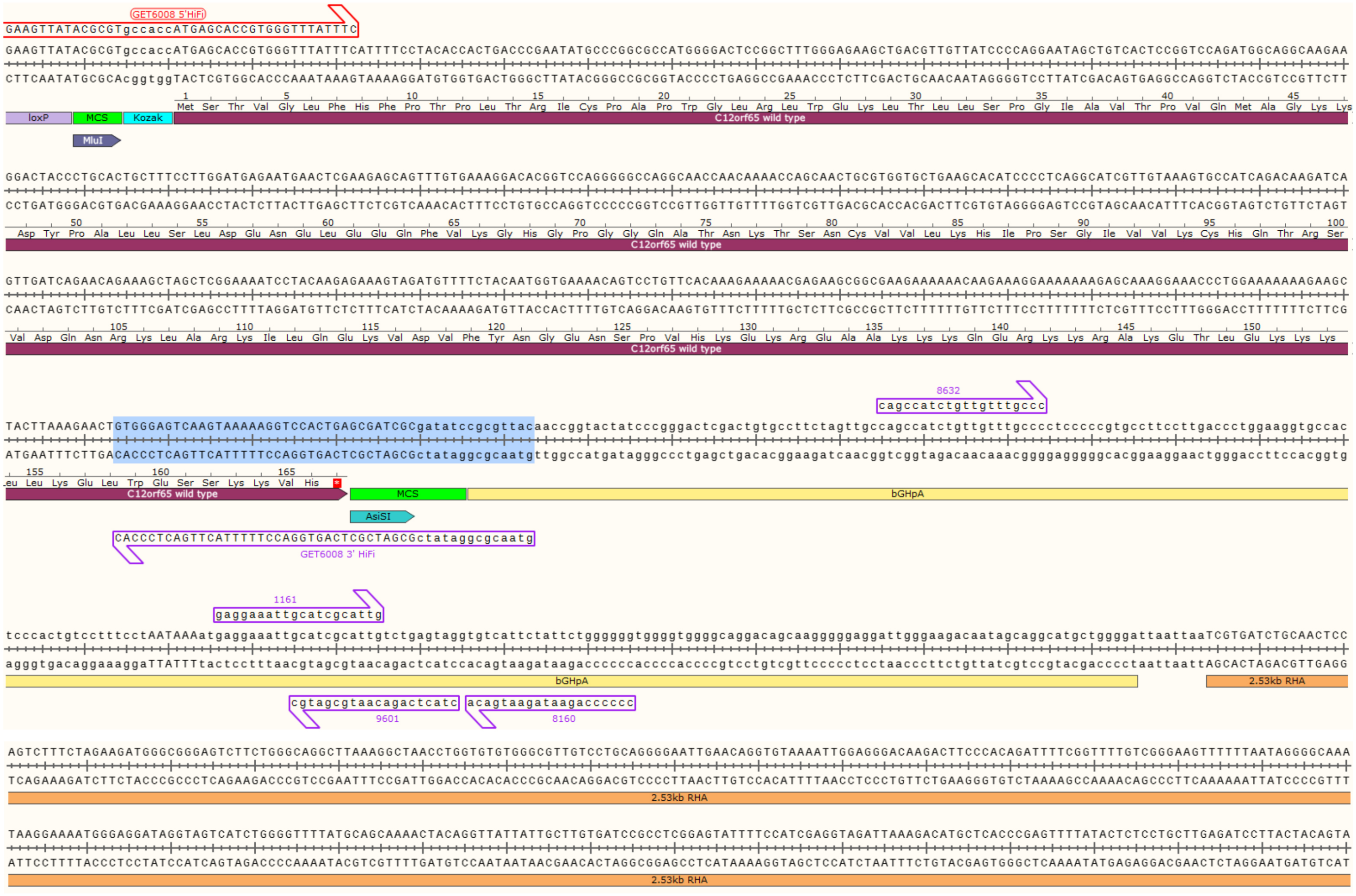

Disease Models & Mechanisms • Supplementary information

Rosa26 KI– Long Range PCR Genotyping

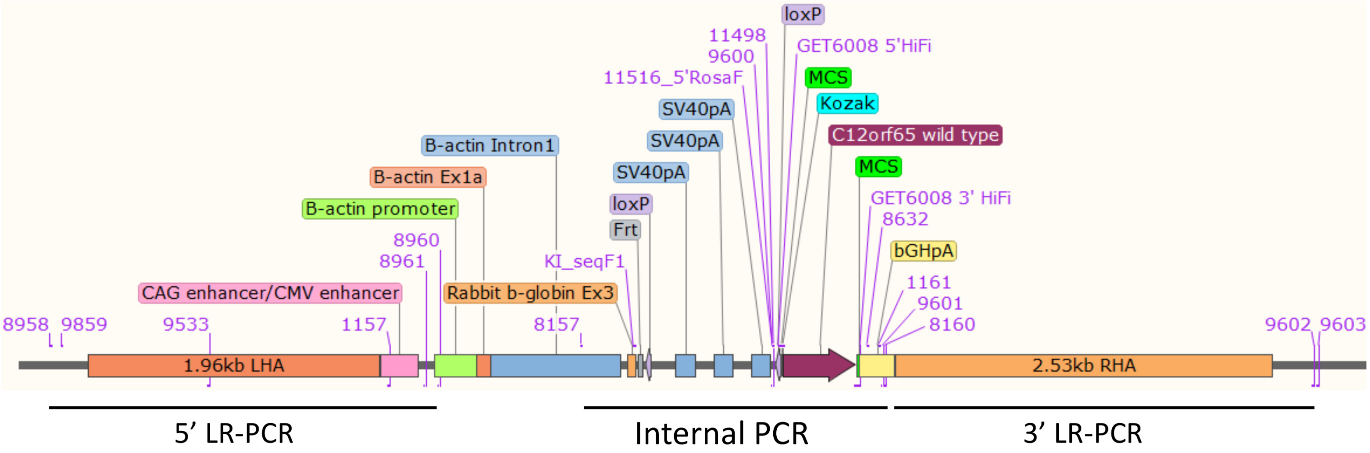

<u>Internal PCR; 2.1kb</u>
8157_pCAG F1: TATGGTAATCGTGCGAGAGG
8160_bGHpA R1: CCCCCCAGAATAGAATGACA

<u>5' LR-PCR; 2.6kb</u>
8958_Lha F1: GAGTCCAAGAATGTGAGGTGG
8959_Lha F2: CTGGAAAACCAGAGGAGAGG
8960_Lha R1: GAGAGTGAAGCAGAACGTGG
8961_Lha R2: GTACTGGGCATAATGCCAGG

<u>3' LR-PCR; 3.0kb</u>
8632_bGHpA F1: CAGCCATCTGTTGTTTGCCC
1161_bGHpA F2: GAGGAAATTGCATCGCATTG
9602_Rha R1: AGAATCTGACCTGCAAGTTCC
9603_Rha R2: CAAGCTCCCTTAGTGTGTCC

Fig. S2. Annotated sequence of transgenic constructs inserted into *Rosa26* locus. Sequence of wild-type transgenic construct inserted into *Rosa26* locus annotated with homology arms, and wildtype human *MTRFR* sequence to create the WTKI-Tg mice. An indentical construct was made for the 3K>A-Tg mice, the only difference being three lysines mutated to alanines (K146, 153, 156 to A).

Disease Models & Mechanisms • Supplementary information

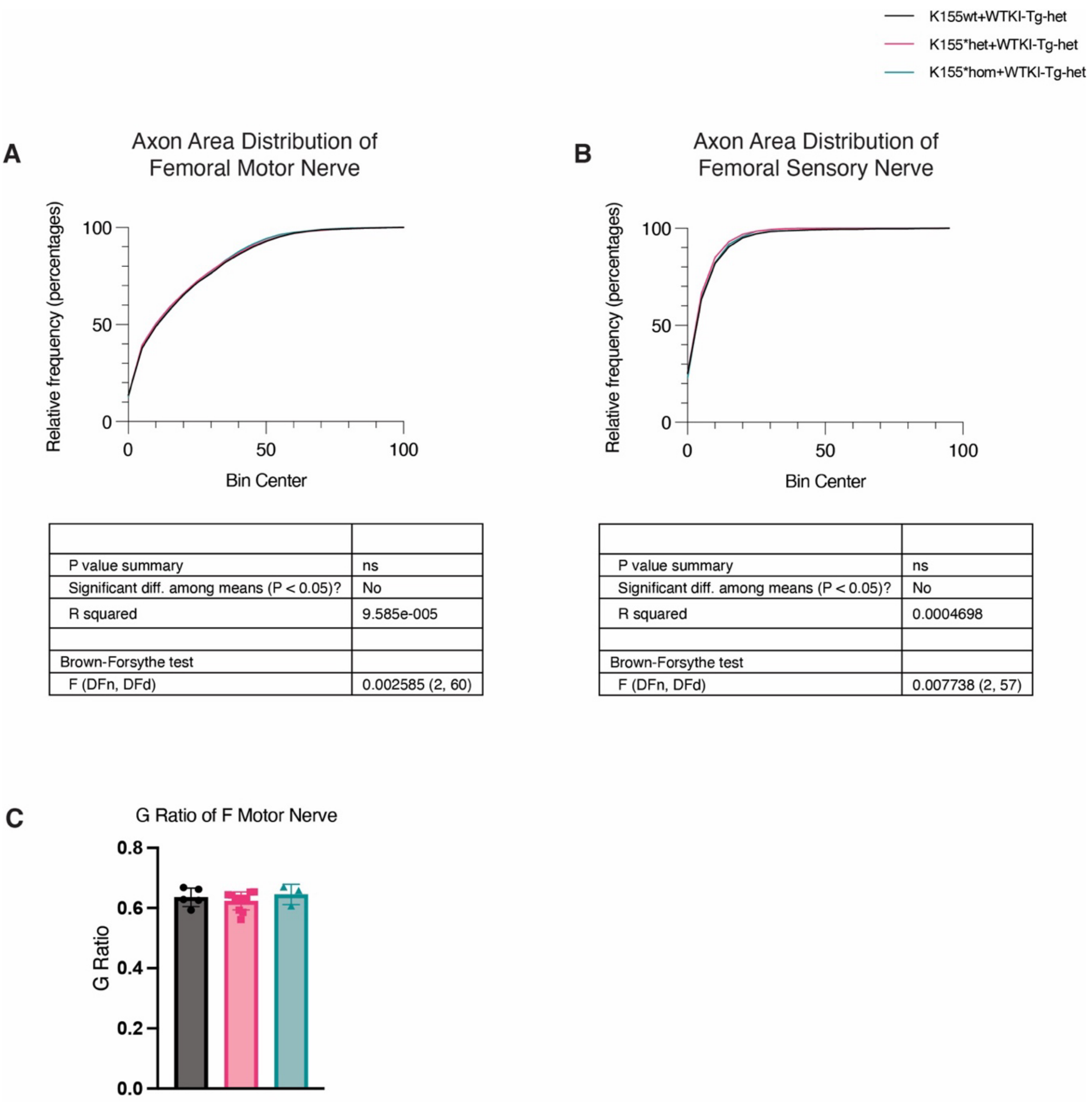

P value summary	ns
Significant diff. among means (P < 0.05)?	No
R squared	9.585e-005
Brown-Forsythe test	
F (DFn, DFd)	0.002585 (2, 60)

P value summary	ns
Significant diff. among means (P < 0.05)?	No
R squared	0.0004698
Brown-Forsythe test	
F (DFn, DFd)	0.007738 (2, 57)

Fig. S3. Rescue of K155* femoral nerve histopathology with heterozygous WTKI-Tg. A and B) Using images taken at 40X of femoral nerves, femoral motor (A) and sensory (B) nerve axon diameters were evaluated. Data are plotted as cumulative histograms showing the distributions of axon sizes were not different in any genotypes. A) Over 500 axons per nerve (538-583) were evaluated, both femoral motor nerves from K155wt n=6, K155*het n=9, K155*hom n=3 were evaluated for these analysis. B) Over 600 axons per nerve (651-868) were evaluated, both femoral sensory nerves from K155wt n=4, K155*het n=12, K155*hom n=4 were evaluated for these analysis. C) G-ratio analysis examining the ratio of myelin thickness to axonal size were determined for over 50 axons per femoral motor nerve. Measurements were taken from the same images of femoral motor nerves as mentioned above. K155wt n=6, K155*het n=9, K155*hom n=3. There were no differences of myelination between the genotypes. Error bars ± S.D. Statistical analysis was performed using one-way ANOVA (A-C).

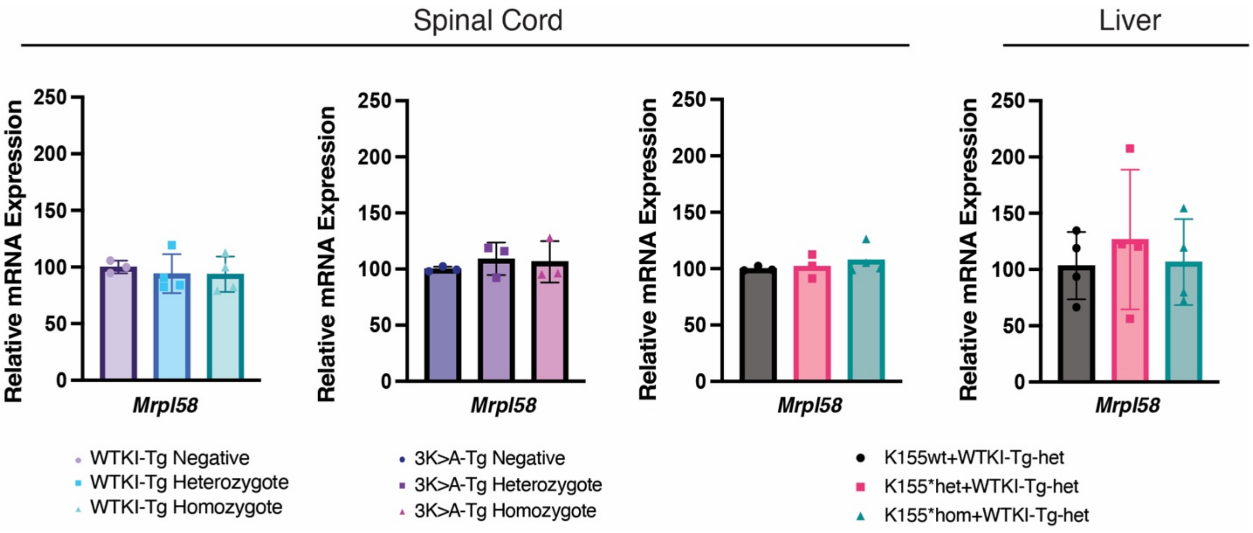

Fig. S4. Presence of MTRFR transgene does not affect endogenous expression of Mrpl58. A) Spinal cord tissues from WTKI-Tg (WTKI-Tg-negative n=3, WTKI-Tg-heterozygous n=4, WTKI-Tg-homozygous n=4), 3K>A-Tg (3K>A-Tg-negative n=3, 3K>A-Tg-heterozygous n=3, 3K>A-Tg-homozygous n=3) and *Mtrfr*^{K155*}+WTKI-Tg-het (K155wt n=3, K155*het n=3, K155*hom n=4) mice was taken for RT-qPCR analysis and there were no deviations from endogenous levels of *Mrpl58*. B) Liver tissue from *Mtrfr*^{K155*}+WTKI-Tg-het (K155wt n=4, K155*het n=4, K155*hom n=4) mice was taken for RT-qPCR analysis and there were no deviations from endogenous levels of *Mrpl58*. Statistical analysis was performed using one-way ANOVA (A and B).

Disease Models & Mechanisms • Supplementary information
